# Methodological study protocol for The European Atlas of clinical trials in cancer and haematology

**DOI:** 10.3389/fphar.2025.1558556

**Published:** 2025-10-14

**Authors:** Mercè Cases, Rachel Giles, Tamás Ágh, Maria Piggin, Jan Geissler, Monica Racovita, Judit Hagymásy, Lora Ruth Wogu, Ákos Józwiak, Albina Hyseni-Bocolli, Dalma Hosszú, Ananda Plate, EuroACT Working Group

**Affiliations:** 1 European Patient Advocacy Institute, Riemerling, Germany; 2 VHL Europa, Vlaardingen, Netherlands; 3 Syreon Research Institute, Budapest, Hungary; 4 PNH Global Alliance, Tiel, Netherlands; 5 Workgroup of European Cancer Patient Advocacy Networks, Barcelona, Spain; 6 Myeloma Patients Europe, Brussels, Belgium; 7 European Sickle Cell Federation-ESCF, Dublin, Ireland; 8 Childhood Cancer International – Europe, Vienna, Austria; 9 University of Pécs Institute of Psychology, Pécs, Hungary

**Keywords:** cancer, haematology, rare disease, patient, patient reported outcome, clinical trials

## Abstract

**Introduction:**

Inequalities in access to clinical trials in cancer, haematology and rare diseases, along with the inconsistent incorporation and reporting of patient reported outcome measures (PROMs) are a long-addressed issue by patient communities. The European Atlas on Clinical Trials in Cancer and Haematology (EuroACT) is a patient-led investigation assessing regional inequalities in access to clinical trials, the frequency and type of patient reported outcome (PRO) data collection in trials, and the reporting of PRO findings in selected solid tumours, malignant/non-malignant haematological conditions and rare diseases across Europe.

**Methods and analysis:**

This protocol outlines the development of three comprehensive datasets [i.e., clinical trials, patient reported outcome and experience measure (PROM, PREM), and publication datasets] along research questions and analysis plan for the EuroACT study. Data for the analysis were sourced from public clinical trial registries (e.g., EudraCT, ClinicalTrials.gov), PRO databases, and published literature, and were subsequently processed in several steps, including standardisation, enrichment, and merging. The analysis plan is organised into three workstreams, each focusing on hypotheses related to the geographical distribution of clinical trials, the use of PROMs in trials, and the frequency of PRO data publication, addressing multiple primary and sub-research questions. The EuroACT study has been co-developed with the patient community, involving a steering group of patient representatives at each step.

**Results:**

A dataset of interventional trials and observational studies with European sites resulted containing 11,185 trials and 1.8 million data points for interventional trials, and 3,723 trials and 2,200 data points for observational studies. The PROM/PREM dataset contains information on 631 PROMs and 14 PREMs. The publication dataset development resulted in a comprehensive dataset containing information on 14,484 scientific publications.

**Discussion:**

The EuroACT research project integrates high-quality data sources, including EudraCT and ClinicalTrials.gov (NCT), with advanced data processing techniques. The data access and processing workflows were developed to enhance transparency, reproducibility, and reliability, while also laying the groundwork for future automation efforts.

## Introduction

Inequalities in access to clinical trials for patients with cancer, haematological and rare diseases have long been a concern within patient communities and are frequently highlighted in healthcare discussions. Systemic healthcare inequalities that likely extend to participation in trials, particularly for rare and haematological cancers. Inequalities in infrastructure, awareness, and socioeconomic conditions remain significant barriers. (Sung et al., 2021; U.S. Food and Drug Administration, 2023).

These disparities are not only negative in terms of access to innovative and sometimes life-saving therapies that are not yet approved, but they have long lasting impact on the infrastructure of a country and the future reimbursement of therapies in those countries. Without clinical trials in a specific country, drugs are not tested within that population, resulting in a lack of localized data, meaning drugs are not tested in diverse populations. Clinicians may have limited exposure to these new treatments, which affects their ability to provide informed guidance, clinical testimony, and advocacy when engaging with regulatory and reimbursement authorities. In addition, evidence has often lacked regarding the lived experiences of patients.

Between 2010 and 2018, the total number of oncology clinical trials in Europe increased by 33%, with a greater relative growth in early-phase trials (phase I–II) compared with late-phase trials (phase II–III). According to Carneiro et al. (2020), when looking at all 18,000+ trials identified in their study, a higher proportion were conducted in Southern and Western Europe (13%–15% of all trials) compared to Central and Eastern Europe (CEE) and Northern Europe, where the proportions ranged from 4% to 9%. Participation in clinical trials offers significant benefits to patients, for example, extra monitoring, additional healthcare, improved clinical outcomes including survival (Unger et al., 2014), and the ability to access novel treatments early. It is equally important for patients to be adequately informed about the impact of how these novel treatments may affect their quality of life beyond disease progression. Transparency in the system and accurate data reporting can significantly improve recruitment, yet many patients remain hesitant due to concerns about risks and uncertainties surrounding clinical trial participation. (Bouzalmate-Hajjaj et al., 2022).

Structural (e.g., small population size, lack of funding, qualified staff), clinical (e.g., narrow eligibility criteria, heterogeneity in clinical practice) or physician and patient barriers (e.g., lack of information) have been reported as barriers to accessing clinical trials. (Myeloma Patients Europe, 2022; International Kidney Cancer Coalition, 2022). Once enrolled, patients may face additional challenges beyond gaining access. A key example is the lack of systematic and meaningful use of PROMs (Patient Reported Outcome Measures) during and after trial participation, which represents a core post-enrolment challenge. Closely linked to this, and beyond issues of poor reporting, many trials still do not systematically integrate PROMs into their design or conduct. Quality of life (QoL) is more multidimensional than the presence or absence of disease-related symptoms typically captured by PRO instruments. Tools currently used in clinical trials often overlook important aspects such as psychological distress, financial burden, caregiver impact, and the logistical strain of participation. PROs are also commonly collected only up to or shortly after treatment discontinuation, leaving long-term effects - such as delayed toxicities - unrecorded. These limitations represent a significant challenge for patients after enrolment in trials: their lived experiences are not systematically captured, potentially diminishing both the relevance of the data and the sense of being seen and supported during participation. The patient community therefore advocates for more comprehensive and longitudinal PRO tools that reflect emotional wellbeing, financial toxicity, caregiver burden, and other real-world concerns, both in trials and in routine care (Hasanov et al., 2022).

Beyond these content limitations, the absence of systematically implemented PROMs contributes to broader gaps in patient follow-up and care quality. PROMs can support improved outcomes by enabling personalised, proactive care, yet many health systems and trial protocols lack mechanisms for consistent use (Campbell et al., 2022). Without structured PROM integration, patient perspectives risk being underrepresented, reducing opportunities for timely intervention and continuity of care. This disconnect persists even when PROs are included: a review of EMA oncology approvals found that although 78% of confirmatory trials contained PRO endpoints, only 17.8% of resulting drug labels reflected this data, pointing to persistent challenges in implementation, data quality, and regulatory translation (Teixeira et al., 2022).

Reporting outcomes is another challenge that suggests further areas of improvement and potential advocacy action points. To address reporting issues in clinical research, several publication and reporting guidelines have been introduced. (European Medicines Agency, 2016; Calvert et al., 2013; Cella et al., 2007). Poor reporting of trial results has significant consequences, including patients lacking the necessary information to make informed decisions. Additionally, inconsistencies within publications lead to missing information in abstracts, poor reporting of adverse events, and selective reporting of trial outcomes, even within primary endpoints, let alone PROMs. (MacCarthy et al., 2018). The patient voice is also underrepresented in reporting, often lacking pre-defined patient reported outcome (PRO) hypotheses, methods for data collection and statistical approaches. (Bylicki et al., 2015). Even when PROs are measured in clinical trials, it does not guarantee that clinicians and patients can access or utilise PRO data. Bylicki et al. reviewed all phase III medical oncology clinical trials published between 2007 and 2011 according to the 2013 PROs CONSORT recommendations. (Bylicki et al., 2015). They found that PROs were mostly reported in secondary manuscripts (29% of the clinical trials). When PROs were reported in the main manuscript, the median percentage of the space allocated to the PROs in the methods section was just 16%. PRO instruments were most frequently a measurement of the patients’ quality of life (71%), symptoms (18%) or both (9%). The instruments used to assess patients’ quality of life were most often disease-specific (58%) or at least cancer-specific (35%). (Bylicki et al., 2015).

To successfully advocate for trial access, systematic PRO usage, and consistent reporting of PRO results, the patient community needs to be equipped with up-to-date evidence that can be converted into learnings and action points for all stakeholders involved. Myeloma Patients Europe pioneered an analysis of barriers and facilitators to clinical trial participation of myeloma patients with a special focus on CEE countries. The results showed that in a 19-year period, only 6% of worldwide myeloma trials included patients from CEE. (Myeloma Patients Europe, 2022).

These findings prompted the Workgroup of European Cancer Patient Advocacy Networks (WECAN) and the European Haematology Community to collaborate on the European Atlas on Clinical Trials in Cancer and Haematology (EuroACT) research project. This initiative aims to gain a comprehensive understanding of the recent clinical trial landscape and inequalities to access. The EuroACT study seeks to evaluate three hypotheses for 27 disease areas including various types of solid tumours, malignant and non-malignant haematological conditions, and rare diseases: i) inequalities in access to clinical trials may exist across diseases and European regions due to uneven trial distribution; ii) when trials are conducted, few collect PRO data or utilise tools that do not effectively capture patient experiences with the disease and treatment; and iii) that even when PRO data are collected, it may not be published. This protocol describes the data sources used, the datasets developed and the data analysis plan for the EuroACT study.

## Materials and methods

### EuroACT working group

The EuroACT Working Group, comprising, 4 with a research background 10 patient advocates and 6 researchers, was established to coordinate the research project. The Working Group members are responsible for: i) determining the research hypotheses and developing research questions; ii) identifying data sources and developing the datasets used for the analysis; iii) preparing the data analysis plan; iv) conducting data analysis; and v) interpreting and disseminating the findings.

The active involvement of patients and patient advocates is a core element of the EuroACT Working Group’s approach. By integrating patient voices at every stage, the Working Group ensures that the study outcomes are both meaningful and relevant to the patient community, ultimately fostering greater impact in improving healthcare decisions and policy.

### Data sources and dataset development

**FIGURE 1:**
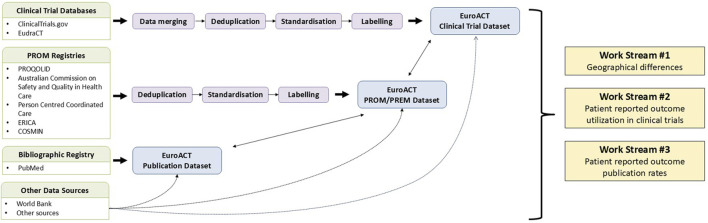
Overview of data integration and analysis workflow for the EuroACT project. Green boxes represent source datasets: Clinical Trial Databases, PROM Registries, Bibliographic Registry, and Other Data Sources. Purple boxes illustrate data processing steps. Blue boxes denote the final integrated datasets. Other data sources contribute supplementing information across all three final datasets as needed. Bi-directional arrows between the blue boxes indicate the interconnected nature of the clinical trial, publication, and PROM/PREM datasets. PREM: patient reported experience measure; PRO: patient reported outcome; PROM: patient reported outcome measure Other sources: Bull et al., 2019; Churruca et al., 2021; de Silva, 2014; Scimagojr, 2024; Clarivate, 2024; World Bank, 2024.

To investigate the research hypotheses outlined at the end of the Introduction, we developed three comprehensive datasets: a clinical trial dataset, a PROM/patient reported experience measure (PREM) dataset, and a publication dataset (Figure 1). These datasets include data on 27 specific disease areas, including acute leukaemia, AL amyloidosis, bladder cancer, brain tumours, breast cancer, chronic lymphocytic leukaemia, chronic myeloid leukaemia, digestive cancers, haemochromatosis, haemophilia, idiopathic thrombocytopenic purpura, kidney cancer, lung cancer, lymphomas, melanoma, myelodysplastic syndromes, myeloma, myeloproliferative neoplasms, neuro-endocrine cancer, pancreatic cancer, paroxysmal nocturnal haemoglobinuria, prostate cancer, sarcoma, sickle cell disease, thalassaemia, thyroid cancer, and Waldenström’s macroglobulinemia. In the following sub-sections, we detail the development of these datasets, including the data sources utilized, the criteria for data inclusion and exclusion, and the methodologies applied to ensure rigorous and comprehensive analysis.

### EuroACT clinical trial dataset

Data on interventional trials and observational studies were sourced from the European Union Drug Regulating Authorities Clinical Trials Database (EudraCT) (European Union Clinical Trials Register, 2023) and the US-based National ClinicalTrials.gov (NCT) (ClinicalTrials, 2023) for the European region, as defined by the World Health Organization (WHO) (World Health Organization, 2024). The EudraCT database includes all interventional clinical trials on medicinal products submitted to the National Competent Authorities of the European Union/European Economic Area (EEA) from 1 May 2004, to 30 January 2023, under Directive 2001/20/EC (EUR-Lex, 2001), as well as trials conducted outside the EEA that are part of a Paediatric Investigation Plan or conducted under Article 45 or 46 of EUR-Lex (2006). The NCT, developed by the United States National Institutes of Health and launched in 2000 as part of the Food and Drug Administration Modernization Act of 1997 (Food and Drug Administration, 1997), provides information on both interventional trials and observational studies.

Data from trials that did not meet any of the exclusion criteria were included, regardless of the intervention. For the NCT database, the exclusion criteria were trials not investigating any of the target conditions; trials ending before 1 January 2017; trials not registered in at least one European country; and phase 1 trials. Due to differences in available data fields between the two registries, the exclusion criteria for the EudraCT database were: trials not investigating any of the target conditions; trials starting before 1 January 2012 (assuming trials registered 5 years before the end date are already over); and phase 1 trials.

The data processing workflow involved several key steps: access and querying, standardisation and enrichment, and merging. The NCT database was accessed via an application programming interface (API), providing the most recent metadata, statistics, and clinical trial information available on NCT. Since EudraCT does not support automated database access, we developed a hypertext markup language (HTML) parser and webpage content extraction pipeline. Queries were carefully compiled for all 27 disease areas of interest. While both registries offer internal synonym searches, these do not always cover every synonym or subclass of a disease area. For example, searching for “digestive cancer” in NCT may include “liver neoplasm” but not its synonym “liver cancer”. To ensure comprehensive searches across all disease categories, we supplemented our queries with Unified Medical Language System (UMLS) (UMLS Knowledge Sources, 2023) condition subcategory terms. The UMLS’s narrower concepts for each disease area applied in the search strings are listed in Supplementary File 1. The original queries conducted in August 2023 produced two datasets: the NCT dataset with 327 data fields and the EudraCT dataset with 344 data fields, each structured differently.

The data processing workflow, including access and querying, standardisation and enrichment, and merging, was implemented using KNIME (version 4.7.4) (Berthold et al., 2009), an open-source platform for data science released under an Open Source GPLv3 license. This platform was chosen for its robust data integration, analysis, and reporting capabilities. In addition to standard KNIME functionalities, the Palladian toolkit (Katz et al., 2024) was employed for data retrieval. Palladian, a Java-based toolkit, offers advanced algorithms for text processing, classification, and extraction of various types of information, which were essential for the successful processing of the clinical trial data.

The datasets required substantial cleaning and enrichment to prepare them for merging and further analysis. These steps included deduplication, filtering relevant data fields, filtering relevant records for the European region, renaming data fields, and handling missing values through data enrichment. Data enrichment involved developing algorithms to find or calculate missing data if information was available in other fields. The most crucial step was data standardisation, ensuring consistent categories across the two datasets, which is vital for successful merging. Standardisation steps were applied to all relevant data fields, resulting in 59 distinct, standardised data fields, listed in Supplementary File 2.

The final step was merging the EudraCT and NCT datasets according to predefined rules. Trials registered in both EudraCT and NCT were linked, with decisions made on which data to use during the merger process. The NCT registry, being more consistent and standardised with fewer missing values, was prioritised during merging. However, merging was carefully defined at the data field level to ensure the best outcomes. The rules for data field-level mapping are detailed in Supplementary File 3. During merging, clinical trials were also filtered for completion status. While the NCT registry allowed querying for completed trials, the EudraCT did not. Therefore, specific rules were introduced to determine which trials were completed, considering inconsistencies in how trial organisers updated statuses across the two databases. The criteria for a “Completed” trial were established as follows: i) trials registered only in EudraCT were considered complete only if all statuses were marked as completed for all countries with sites; ii) for trials registered in both EudraCT and NCT registries, all statuses had to be marked as completed for all countries with sites, or at least one country had to have a completed status in EudraCT, with EudraCT results present; or iii) trials registered only in NCT were deemed completed. In all other cases, the trial was considered “Not completed”.

It is also important to note that only the NCT database provides detailed information on trial site locations, including longitude and latitude geographical data. Due to the lack of consistent trial site names, this geographic data is essential for accurately identifying precise trial locations. Unfortunately, the EudraCT database does not yet include geographical information on trial site locations. However, this information is expected to be incorporated into the EudraCT database with the new version that will become mandatory by 2025, with initial data collection having begun in January 2022. Given this limitation, the trial site locations dataset for this study was limited exclusively to data derived from the NCT database.

The final EuroACT clinical trial dataset was provided in Microsoft (MS) Excel format, enabling the development of a structured data model tailored for analysis. This model established relationships between data pairs to support data cleaning and grouping, specifying data formats for each column (e.g., string, number, Boolean) and defining relationships (one-to-many, many-to-many). Given that the database listed multiple entries for the same trial corresponding to the number of European countries involved, it was essential to precisely identify each trial. A unique identifier for each trial was constructed using the cryptographic hash (MD5 algorithm) of the NCT and EudraCT identifiers (ID).

### EuroACT PROM/PREM dataset

As a first step, a targeted literature review of systematic literature reviews (SLRs) and meta-analyses (MAs) was conducted to identify PROMs and PREMs used across the 27 disease areas under investigation. A literature search was performed in MEDLINE (via PubMed) to identify relevant systematic literature reviews and meta-analyses published between 2018 and 2022, with no geographical restrictions applied. Search terms for the 27 disease areas, as well as those related to PROMs and PREMs, were defined using the Meta thesaurus of the UMLS database. (UMLS Knowledge Sources, 2023). Distinct search strings were developed for each disease area, combining a consistent string for PROMs and PREMs with a unique string tailored to the specific disease area. (Supplementary File 4) Publications were deemed eligible for inclusion in the review if they met the following criteria: included patients with one of the specified disease areas, reported data related to PROMs or PREMs, were designed as a systematic literature review or meta-analysis, published between 2018 and 2022, and written in English. Articles which did not report data related to PROMs or PREMs were excluded. A total 161,186 articles were identified in the literature search. Of these 4,356 were SLR and/or MA. The number of SLR and/or MA articles published in English between 2018 and 2022 was 2,280. Detailed search results by disease area can be found in Table 1.

**TABLE 1:** Number of search hits.

Disease area	Number of search hits related to the disease area	Number of search hits related to the humanistic burden	Combined search hits	Number of combined search hits limited to SLR/MA	Number of combined search hits limited to SLR/MA, published in the past 5 years	Number of combined search hits limited to SLR/MA, published in the past 5 years, written in English	Proportion of SLR/MA	Proportion of English SLR/MA in past 5 years
Acute leukaemia	24 627	1 803 416	1 292	19	6	6	1.5%	0.5%
AL amyloidosis	309 322	1 803 098	14 868	345	219	215	2.3%	1.4%
Bladder cancer	42 462	1 803 138	2 893	129	78	77	4.5%	2.7%
Brain tumours	187 005	1 803 138	12 393	304	166	166	2.5%	1.3%
Breast cancer	427 333	1 777 885	35 456	1 208	635	627	3.4%	1.8%
Chronic lymphocytic leukaemia	32 684	1 803 018	2 029	20	8	8	1.0%	0.4%
Chronic myeloid leukaemia	34 118	1 803 018	1 676	24	11	11	1.4%	0.7%
Digestive cancers	11 345	1 803 416	1 230	52	35	35	4.2%	2.8%
Haemochromatosis	12 754	1 803 416	422	9	3	3	2.1%	0.7%
Haemophilia	22 642	1 803 416	2 481	54	24	24	2.2%	1.0%
Idiopathic thrombocytopenic purpura	4 612	1 803 416	150	3	2	2	2.0%	1.3%
Kidney cancer	18 052	1 803 416	1 160	46	29	29	4.0%	2.5%
Lung cancer	200 910	1 803 416	15 472	576	278	276	3.7%	1.8%
Lymphomas	262 638	1 803 416	11 612	187	91	90	1.6%	0.8%
Melanoma	134 805	1 803 416	6 944	130	71	71	1.9%	1.0%
Myelodysplastic syndromes	31 895	1 805 657	2 000	39	23	23	2.0%	1.2%
Myeloma	69 007	1 803 098	4 295	79	35	35	1.8%	0.8%
Myeloproliferative neoplasms	59 596	1 803 417	2 644	29	13	13	1.1%	0.5%
Neuro-endocrine cancer	196 111	1 803 098	8 104	134	78	78	1.7%	1.0%
Pancreatic cancer	52 157	1 803 018	4 175	119	68	68	2.9%	1.6%
Paroxysmal nocturnal haemoglobinuria	4 009	1 803 098	234	3	1	1	1.3%	0.4%
Prostate cancer	145 051	1 803 098	15 427	525	267	263	3.4%	1.7%
Sarcoma	190 926	1 803 018	5 999	85	37	37	1.4%	0.6%
Sickle cell disease	40 298	1 803 099	3 243	116	63	63	3.6%	1.9%
Thalassaemia	31 330	1 803 099	1 839	37	20	20	2.0%	1.1%
Thyroid cancer	68 303	1 803 099	2 979	78	36	36	2.6%	1.2%
Waldenström’s macroglobulinemia	7 259	1 803 255	169	6	3	3	3.6%	1.8%

MA, meta-analysis; SLR, systematic literature review.

As a second step, we developed a comprehensive repository of 2,099 PROMs and PREMs by merging data from eight publicly available PROM and/or PREM databases. (PROQOLID, 2022; Safety and Quality, 2022; P3c, 2022; Bull et al., 2019; Churruca et al., 2021; de Silva, 2014; ERICA, 2022; COSMIN, 2022). The repository includes data on the short and full names of the instruments, type of instrument (i.e., PROM or PREM), category of instrument (i.e., generic or disease-specific), condition/status measured, and data source.

Next, a literature screening process was applied using R software (version 4.2.2.). This process involved developing regular expressions for pattern matching with text strings, allowing us to identify references to PROMs and PREMs within the included full-text records. The regular expressions were based on the short and full names of the instruments in the repository, with variations that account for common abbreviations and naming conventions. Full-text articles were converted from PDFs to text files using the ‘pdftools package’, which extracted the textual content of each document for further analysis. Following this, text cleaning and context extraction were performed using the ‘qdap package’. The developed regular expressions were then used to search the text files using the ‘stringr package’ to identify occurrences of the PROMs and PREMs. This search included counting the occurrences of each regular expression and identifying instances where multiple expressions occur within proximity, suggesting a strong match. Finally, the potentially relevant PROMs and PREMs identified through this automated process were validated by manual search of full texts to ensure accuracy and relevance. In total, 2,235 publications were included in the data extraction. Identified PROMs or PREMs from the reviewed publications were included in the PROM/PREM dataset with information on short name of the instrument, full name of the instrument, type of instrument (i.e., PROM or PREM), specificity of instrument (i.e., generic or specific), condition/status measured. No PROMs or PREMs were excluded; all instruments identified were included in the dataset.

### EuroACT publication dataset

To develop the third dataset, we conducted a systematic search to identify publications associated with the clinical trials included in our clinical trial dataset. Unique trial identifiers from both the EudraCT and NCT databases were extracted. Using the ‘RISmed’ package in R software (version 4.2.2), we performed two types of searches for each trial ID: a standard search using the secondary identifier tag and a comprehensive search using the ‘Title/Abstract’ tag. Metadata for each identified publication, including PubMed ID (PMID), article title, abstract, journal abbreviation, journal full name, PubMed Central^®^ (PMC) identifier, and keywords, were extracted and compiled. The resulting data were then de-duplicated, standardised, and merged to create a final dataset that links each trial ID to its corresponding publications.

### Research questions and data analysis plan development

According to the research hypotheses, the research questions and data analysis plan were developed through a structured, collaborative process to address geographical distribution of clinical trials, usage of PROMs in clinical trials, and the frequency of publishing PRO data. Research questions for each area were co-developed by researchers and patient representatives during an online 3-h workshop held on 13 February 2024, followed by an iterative refinement process within the EuroACT Working Group. The data analysis steps were specifically designed to address these research questions and will involve descriptive statistics, with results planned to be visualized through an online dashboard system.

## Results

### EuroACT datasets

As a result of applying the dataset development procedures described above, we obtained a merged, comprehensive dataset of interventional trials and observational studies with European sites, containing 11,185 trials and 1.8 million data points for interventional trials, and 3,723 trials and 2,200 data points for observational studies. For the PROM/PREM data, we obtained a comprehensive dataset containing information on 631 PROMs and 14 PREMs. The publication dataset development resulted in a comprehensive dataset containing information on 14,484 scientific publications (original queries conducted on 16 June 2024). The datasets were designed to serve further data analysis purposes.

### EuroACT research questions and data analysis plan

The data analysis for this research is structured around three core work streams, each aligned with specific research hypotheses: i) geographical distribution of clinical trials; ii) usage of PROMs in clinical trials; and iii) frequency of publishing PRO data. Each work stream was divided into 5, 4, and 3 primary research focuses, respectively, with research questions to provide more granularity, co-developed by researchers and patient representatives (Table 2). In total, 58 research questions were developed, and the full list is provided in Supplementary File 5.

**TABLE 2:** Research focuses of EuroACT research project.

Work stream	Research focus	Objective
Work Stream 1: Geographical distribution of clinical trials	Regional and country differences in clinical trial activities across disease areas	To analyse and compare the number of clinical trials and clinical trial sites across different regions of Europe and within countries, covering all disease areas
	Influence of socioeconomic and scientific factors on country differences	To examine the impact of selected socioeconomic and scientific factors on the variation in the number of clinical trials and clinical trial sites across countries
	Impact of study population age group on country differences	To explore how the age group of the study population (adults vs. children/young adults) influences the number of clinical trials and clinical trial sites across countries
	Influence of study sponsor type on regional and country differences	To explore how the study sponsor type (commercial vs. non-commercial) influences the number of clinical trials and clinical trial sites across regions and countries
	Influence of disease rarity on regional and country differences	To assess how the rarity of a disease affects the distribution of clinical trials and clinical trial sites across different regions of Europe and within countries
Work Stream 2: Usage of PROMs in clinical trials	Geographic variability in PROMs utilization in clinical trials across disease areas	To assess and compare the utilisation of PROMs in clinical trials across various European regions and countries, covering all disease areas
	Influencing factors of PROMs usage in clinical trials	To examine the impact of selected factors on PROMs usage in clinical trials, considering all disease areas collectively
	Nature of outcomes assessed by PROMs in clinical trials	To evaluate the characteristics of PROMs used in clinical trials, covering all disease areas collectively and each individual disease area
	Characterizing PROMs utilized in clinical trials	To evaluate the types of PROMs used to measure in clinical trials, covering all disease areas collectively and each individual disease area
Work Stream 3: Frequency of published PRO data	Publication rate of PROs in clinical trials across disease areas	To evaluate the rate at which PRO results from clinical trials are published, covering both a collective analysis across all disease areas and detailed analyses for each individual disease area
	Influence of clinical trial characteristics on PRO publication rate	To assess the relationship between selected clinical trial characteristics and the publication rate of PRO results, considering all disease areas collectively
	Characterising publications reporting PRO data	To assess the publication types, access levels, and scientific impact of articles reporting PRO data from clinical trials across all disease areas and individual ones

PRO, patient reported outcome; PROM, patient reported outcome measure.

For certain questions, the investigated disease areas were separated into malignant and non-malignant disorders. Malignant disorders include acute leukaemia, neuro-endocrine cancer, AL amyloidosis, pancreatic cancer, bladder cancer, kidney cancer, brain tumours, lung cancer, prostate cancer, breast cancer, lymphoma, sarcoma, chronic lymphocytic leukaemia, melanoma, chronic myeloid leukaemia, myelodysplastic syndromes, myeloma, thyroid cancer, digestive cancers, myeloproliferative neoplasms and Waldenström’s macroglobulinemia. Non-malignant disorders include haemophilia, idiopathic thrombocytopenic purpura, paroxysmal nocturnal haemoglobinuria, sickle cell disease, thalassaemia and haemochromatosis.

For analysis, the data conversion will be performed using Python (version 3.9.13) on the clinical trials, PROM/PREM, and publication datasets developed for the EuroACT project, enriched with external data from relevant sources. (Scimagojr, 2024; Clarivate, 2024; World Bank, 2024). The previously acquired datasets will be transformed into a relational format using an entity-relationship model, ensuring data integrity, deduplication, and adherence to good data management practices. The entity-relationship model defines data points and their interrelationships, such as one-to-many (e.g., one trial can have multiple phases) and many-to-many (e.g., multiple trials can share the same trial site locations), thereby preventing data duplication. To uniquely identify trials, a single unique identifier will be generated using a cryptographic hash (MD5) of the NCT and EudraCT identifiers.

For visualisation, a dashboard system will be developed using Apache Superset™ (version 3.1.0). (The Apache Software Foundation, 2024). This system will translate structured query language (SQL) queries into dynamic, interactive charts, organised into dashboards that represent different research areas. The process will include multiple stages, from SQL query development to chart integration, ensuring accurate and effective data presentation. The dashboard system will also provide advanced features such as user role-based access control, data export options, and real-time data updates, creating a flexible environment for data analysis and reporting. These functionalities will enhance analysis efficiency, support in-depth data exploration, and facilitate clear communication of research findings.

## Discussion

The EuroACT study protocol development demonstrates the importance of high-quality databases to address research questions concerning clinical trial accessibility and patient-reported outcomes in Europe. The study’s methodological rigor in data collection, standardization, and integration represents a significant contribution to the field of health data science, particularly in the context of oncology, haematology and rare disease clinical research. In addition to rigorous database building, a key strength of this study lies in its patient-centred design. By originating from the patient community and involving patient representatives at every stage - from hypothesis generation to dissemination - the EuroACT research project ensures that its outcomes resonate with the needs of the patient community. This participatory approach underscores the critical role of patient advocacy in shaping research agendas and addressing systemic barriers in clinical trial access.

The study relies on two robust clinical trial data sources: NCT and EudraCT. The comprehensive nature of these datasets allowed for a wide-ranging exploration of trial characteristics, including trial design, geographical distribution, and reporting practices. However, integrating these datasets presented unique challenges due to differences in data structures, terminology, and completeness. To address these challenges, the study employed advanced data processing techniques, including data standardization and enrichment, and leveraged tools such as KNIME and R. These efforts ensured that datasets were harmonized for analysis, providing a coherent and enriched view of the clinical trial landscape in Europe.

In addition to the clinical trial dataset, the development of the PROM/PREM dataset reflects a robust and innovative approach to synthesizing patient-reported outcome measures data. By merging information from eight publicly available PROM/PREM repositories with systematic literature reviews, the study created a comprehensive repository of PROMs and PREMs, categorized by type, specificity, and condition measured. The use of text-mining algorithms and regular expressions to identify relevant measures in full-text articles demonstrates the potential of automated tools to enhance the efficiency and accuracy of dataset construction.

The publication dataset adds another dimension to this research, linking clinical trials to their associated publications using metadata extraction and unique trial identifiers. This linkage allows for the analysis of publication rates and the completeness of PRO reporting, revealing systemic gaps in the dissemination of patient-centred data.

Despite these achievements, the database-building process was not without limitations. Variability in the depth and accuracy of reporting between NCT and EudraCT introduced challenges in ensuring data consistency. For example, discrepancies in trial statuses and incomplete fields required the application of predefined rules to manage differences, which may have introduced minor biases. Also, the absence of detailed trial site data in the EudraCT database restricts our ability to map trial accessibility comprehensively. Furthermore, the reliance on publicly available data inherently excludes unpublished trial results, potentially underestimating trial activities in underrepresented regions.

The EuroACT study protocol development highlights the critical need for harmonized data reporting standards across global and regional registries. This includes improving the granularity of geographic and demographic information, as well as mandating the inclusion of PRO-related data in trial registries. The integration of such enhancements would further support large-scale data analyses and promote equity in clinical trial access across diverse populations. The EuroACT study demonstrates how systematic database construction can not only inform research questions but also drive broader policy and advocacy efforts. By providing a comprehensive and accessible resource, the study lays the groundwork for future investigations and underscores the role of robust data systems in advancing patient-centred clinical research.

The EuroACT dashboard system will be publicly accessible to interested parties, with promotion and support provided by the Workgroup of European Cancer Patient Advocacy Networks (WECAN) and European Haematology community. The findings of the study will be disseminated through various channels, including publications (manuscripts in peer-reviewed journals), presentations at scientific conferences, patient advocacy events, lay summaries, policy events both at national and EU levels, and meetings with different stakeholders. Efforts will be made to tailor the communication of results to meet the needs of diverse audiences, including providing accessible summaries for patients and clear, actionable insights for policymakers.

The results of our study should be considered in the light of the following limitations. Variability in data availability and reporting standards across EudraCT and NCT registries may introduce inconsistencies in the analysis datasets. Potential selection bias in the data sources to be used for analysis may disproportionately represent trials from regions with more robust reporting infrastructure or higher participation rates in registries, this could result in underrepresentation of less developed European regions.

In conclusion, the EuroACT study represents a significant step forward in understanding and addressing inequalities in clinical trial accessibility, as well as the usage and reporting of PROMs in clinical trials across Europe, particularly in solid tumours, malignant and non-malignant haematological conditions, and rare diseases. Through the development of comprehensive datasets and the application of rigorous methodologies for data integration, standardization, and analysis, the study will provide essential insights into these critical areas. The active involvement of patient representatives through the whole research process ensures that the outcomes align with the needs of the patient community, fostering greater relevance and impact. Looking ahead, the EuroACT dashboard system and findings will serve as invaluable resources for researchers, policymakers, and advocates, driving evidence-based decisions to improve patient-centred research and healthcare outcomes across Europe.

## Data Availability

The raw data supporting the conclusions of this article will be made available by the authors, upon reasonable request.
