# Correction: Methodological study protocol for the European Atlas of clinical trials in cancer and haematology

**DOI:** 10.3389/fphar.2025.1743351

**Published:** 2025-11-21

**Authors:** Mercè Cases, Rachel Giles, Tamás Ágh, Maria Piggin, Jan Geissler, Monica Racovita, Judit Hagymásy, Lora Ruth Wogu, Ákos Józwiak, Albina Hyseni-Bocolli, Dalma Hosszú, Ananda Plate, EuroACT Working Group

**Affiliations:** 1 European Patient Advocacy Institute, Riemerling, Germany; 2 VHL Europa, Vlaardingen, Netherlands; 3 Syreon Research Institute, Budapest, Hungary; 4 PNH Global Alliance, Tiel, Netherlands; 5 Workgroup of European Cancer Patient Advocacy Networks, Barcelona, Spain; 6 Myeloma Patients Europe, Brussels, Belgium; 7 European Sickle Cell Federation-ESCF, Dublin, Ireland; 8 Childhood Cancer International – Europe, Vienna, Austria; 9 University of Pécs Institute of Psychology, Pécs, Hungary

**Keywords:** cancer, haematology, rare disease, patient, patient reported outcome, clinical trials

Authors **Judit Hagymásy**, **Ákos Józwiak** and **Dalma Hosszú** were erroneously assigned to affiliation 4 PNH Global Alliance, Tiel, Netherlands. This affiliation has now been removed for author Judit Hagymásy, Ákos Józwiak and Dalma Hosszú.

**Affiliation 3** Syreon Research Institute, Budapest, Hungary was omitted for authors Judit Hagymásy, Ákos Józwiak and Dalma Hosszú. This affiliation has now been added for authors Judit Hagymásy, Ákos Józwiak and Dalma Hosszú.

The collaborative group **EuroACT Working Group** was erroneously not credited with authorship in the published article. The consortium should be credited with authorship.

The list of members of the EuroACT Working Group was omitted from the published article. The section Group Members of the EuroACT Working Group has been added to the article as follows: Attila Imre, Anna Bögös, Natacha Bolaños, Susanna Leto di Priolo, Kate Morgan.

The original article has been updated.

