# Supplementary material for: Methodological study protocol for The European Atlas of clinical trials in cancer and haematology
Source: Front Pharmacol. 2025 Oct 14;16:1558556. doi: 10.3389/fphar.2025.1558556 (PMC12580590; doi:10.3389/fphar.2025.1558556)
Supplement: Supplementary file 4 [file Supplementaryfile4.docx]

**Supplement 4. Search strings related to PROMs and the investigated 27 disease areas**

**Table 1. Search string related to PROMs**

| ("patient reported outcome"[Title/Abstract] OR "patient reported outcomes"[Title/Abstract] OR "patient-reported outcomes"[Title/Abstract] OR "patient-reported outcome"[Title/Abstract] OR "outcome, patient reported"[Title/Abstract] OR "outcome, patient-reported"[Title/Abstract] OR "patient-reported outcome (pro)"[Title/Abstract] OR "pro"[Title/Abstract] OR "patient reported outcome measure"[Title/Abstract] OR "patient reported outcome measures"[Title/Abstract] OR "patient-reported outcome measures"[Title/Abstract] OR "patient-reported outcome measure"[Title/Abstract] OR "pro_measures"[Title/Abstract] OR "proms"[Title/Abstract] OR "prom"[Title/Abstract] OR "patient‐reported experience"[Title/Abstract] OR "patient reported experience"[Title/Abstract] OR "patient‐reported experiences"[Title/Abstract] OR "patient reported experiences"[Title/Abstract] OR "pre"[Title/Abstract] OR "patient‐reported experience measure"[Title/Abstract] OR "patient reported experience meausre"[Title/Abstract] OR "patient‐reported experience measures"[Title/Abstract] OR "patient reported experience meausres"[Title/Abstract] OR "prems"[Title/Abstract] OR "patient reported outcome instruments"[Title/Abstract] OR "patient-reported survey instrument"[Title/Abstract] OR "patient outcome"[Title/Abstract] OR "patient outcome assessment"[Title/Abstract] OR "patient outcome assessments"[Title/Abstract] OR "patient outcomes assessment"[Title/Abstract] OR "assessment, patient outcome"[Title/Abstract] OR "assessment, patient outcomes"[Title/Abstract] OR "assessments, patient outcome"[Title/Abstract] OR "outcome assessment, patient"[Title/Abstract] OR "outcomes assessments, patient"[Title/Abstract] OR "outcome assessments, patient"[Title/Abstract] OR "patient centered outcomes research"[Title/Abstract] OR "patient-centered outcomes research"[Title/Abstract] OR "research, patient-centered outcomes"[Title/Abstract] OR "symptom assessment"[Title/Abstract] OR "symptom assessments"[Title/Abstract] OR "assessment, symptom"[Title/Abstract] OR "assessments, symptom"[Title/Abstract] OR "symptom scale"[Title/Abstract] OR "symptom measure"[Title/Abstract] OR "quality of life"[Title/Abstract] OR "life quality"[Title/Abstract] OR "qol"[Title/Abstract] OR "health-related quality of life"[Title/Abstract] OR "health related quality of life"[Title/Abstract] OR "hrqol"[Title/Abstract] OR "health-related quality of life (hrqol)"[Title/Abstract] OR "patient-centered care"[Title/Abstract] OR "patient centered care"[Title/Abstract] OR "person-centered care"[Title/Abstract] OR "person centered care"[Title/Abstract] OR "person-centered cares"[Title/Abstract] OR "care patient centered"[Title/Abstract] OR "cares centered patient"[Title/Abstract] OR "care, patient-centered"[Title/Abstract] OR "care, person-centered"[Title/Abstract] OR "cares, person-centered"[Title/Abstract] OR "health status indicators"[Title/Abstract] OR "health status index"[Title/Abstract] OR "health status indexes"[Title/Abstract] OR "health status indicator"[Title/Abstract] OR "indicator, health status"[Title/Abstract] OR "indicators, health status"[Title/Abstract] OR "health status indices"[Title/Abstract] OR "index, health status"[Title/Abstract] OR "indexes, health status"[Title/Abstract] OR "indices, health status"[Title/Abstract] OR "functional capacity"[Title/Abstract] OR "capacities functional"[Title/Abstract] OR "capacity functional"[Title/Abstract] OR "functional status"[Title/Abstract] OR "status functional"[Title/Abstract] OR "status, functional"[Title/Abstract] OR "physical function"[Title/Abstract] OR "physical functions"[Title/Abstract] OR "function physical"[Title/Abstract] OR "functioning physical"[Title/Abstract] OR "mental function"[Title/Abstract] OR "mental functions"[Title/Abstract] OR "functioning mental"[Title/Abstract] OR "mental functions, unspecified"[Title/Abstract] OR "patient satisfaction score"[Title/Abstract] OR "patient satisfaction score (assessment scale)"[Title/Abstract] OR "patient satisfaction score (observable entity)"[Title/Abstract] OR "patsat - patient satisfaction score"[Title/Abstract] OR "patient self-report"[Title/Abstract] OR "self report"[Title/Abstract] OR "self-report"[Title/Abstract] OR "subject-reported outcome (sro)"[Title/Abstract]) OR (patient reported outcome[MeSH Terms] OR patient reported outcomes[MeSH Terms] OR patient-reported outcomes[MeSH Terms] OR patient-reported outcome[MeSH Terms] OR outcome, patient reported[MeSH Terms] OR outcome, patient-reported[MeSH Terms] OR patient reported outcome measure[MeSH Terms] OR patient reported outcome measures[MeSH Terms] OR patient outcome assessment[MeSH Terms] OR patient outcome assessments[MeSH Terms] OR patient outcomes assessment[MeSH Terms] OR assessment, patient outcome[MeSH Terms] OR assessment, patient outcomes[MeSH Terms] OR assessments, patient outcome[MeSH Terms] OR outcome assessment, patient[MeSH Terms] OR outcomes assessments, patient[MeSH Terms] OR outcome assessments, patient[MeSH Terms] OR patient centered outcomes research[MeSH Terms] OR research, patient-centered outcomes[MeSH Terms] OR symptom assessment[MeSH Terms] OR symptom assessments[MeSH Terms] OR assessment, symptom[MeSH Terms] OR assessments, symptom[MeSH Terms] OR quality of life[MeSH Terms] OR life quality[MeSH Terms] OR health-related quality of life[MeSH Terms] OR health related quality of life[MeSH Terms] OR hrqol[MeSH Terms] OR patient-centered care[MeSH Terms] OR person-centered care[MeSH Terms] OR person centered care[MeSH Terms] OR person-centered cares[MeSH Terms] OR care, patient-centered[MeSH Terms] OR care, person-centered[MeSH Terms] OR cares, person-centered[MeSH Terms] OR health status indicators[MeSH Terms] OR health status index[MeSH Terms] OR health status indexes[MeSH Terms] OR health status indicator[MeSH Terms] OR indicator, health status[MeSH Terms] OR indicators, health status[MeSH Terms] OR health status indices[MeSH Terms] OR index, health status[MeSH Terms] OR indexes, health status[MeSH Terms] OR indices, health status[MeSH Terms] OR functional status[MeSH Terms] OR status, functional[MeSH Terms]) |
| --- |

**Table 2. Literature search strategy of Acute leukaemia**

| Disease area | ACUTE LEUKAEMIA |
| --- | --- |
| Search date | **2022.12.07.** |
| Search terms | ("Acute leukemia"[Title/Abstract] OR "LEUKEMIA ACUTE"[Title/Abstract] OR "Acute leukemia, disease (disorder)"[Title/Abstract] OR "Acute leukemia (morphologic abnormality)"[Title/Abstract] OR "Acute leukemia, disease"[Title/Abstract] OR "Acute leukemia, morphology, including blast cell OR undifferentiated leukemia"[Title/Abstract] OR "Acute leukaemia"[Title/Abstract] OR "Acute leukaemia, disease"[Title/Abstract] OR "Acute leukaemia, morphology, including blast cell OR undifferentiated leukaemia"[Title/Abstract] OR "Acute leukemias"[Title/Abstract] OR "Leukaemia of unspecified cell type, acute"[Title/Abstract] OR "Acute leukaemia NOS"[Title/Abstract] OR "Acute leukemia NOS"[Title/Abstract] OR "Leukaemia acute"[Title/Abstract] OR "Leukemia of unspecified cell type, acute"[Title/Abstract] OR "Acute leukaemia of unspecified cell type"[Title/Abstract] OR "Acute leukemia of unspecified cell type"[Title/Abstract] OR "acute leukemia (diagnosis)"[Title/Abstract] OR "acute leukemia not otherwise specified"[Title/Abstract] OR "stem cell acute leukemia"[Title/Abstract] OR "leukemia, acute"[Title/Abstract] OR "acute leukemia, NOS"[Title/Abstract] OR "acute leukaemias"[Title/Abstract] OR "leukemia; acute"[Title/Abstract] OR "acute; leukemia"[Title/Abstract] OR "Acute leukaemia, NOS"[Title/Abstract] |

**Table 3. Literature search strategy of AL amyloidosis**

| Disease area | AL AMYLOIDOSIS |
| --- | --- |
| Search date | **2022.12.06.** |
| Search terms | ("Primary amyloidosis"[Title/Abstract] OR "Immunoglobulin Light-chain Amyloidosis"[Title/Abstract] OR "Primary Systemic Amyloidosis"[Title/Abstract] OR "AL Amyloidosis"[Title/Abstract] OR "Amyloidosis, Primary"[Title/Abstract] OR "Amyloidosis, Immunoglobulin Light-chain"[Title/Abstract] OR "Amyloidoses, Primary"[Title/Abstract] OR "Primary Amyloidoses"[Title/Abstract] OR "AL Amyloidoses"[Title/Abstract] OR "Amyloidoses, Primary Systemic"[Title/Abstract] OR "Amyloidosis, Immunoglobulin Light chain"[Title/Abstract] OR "Amyloidosis, Primary Systemic"[Title/Abstract] OR "Immunoglobulin Light chain Amyloidosis"[Title/Abstract] OR "Immunoglobulin Light-chain Amyloidoses"[Title/Abstract] OR "Primary Systemic Amyloidoses"[Title/Abstract] OR "Systemic Amyloidoses, Primary"[Title/Abstract] OR "Systemic Amyloidosis, Primary"[Title/Abstract] OR "Amyloid light-chain amyloidosis (disorder)"[Title/Abstract] OR "Primary amyloidosis of light chain type"[Title/Abstract] OR "Amyloid light-chain amyloidosis"[Title/Abstract] OR "AMYLOIDOSIS, SYSTEMIC"[Title/Abstract] OR "AL"[Title/Abstract] OR "primary amyloidosis (diagnosis)"[Title/Abstract] OR "amyloidosis primary"[Title/Abstract] OR "idiopathic amyloidosis"[Title/Abstract] OR "Light-chain amyloidosis"[Title/Abstract] OR "AMYLOIDOSIS, IDIOPATHIC"[Title/Abstract]) OR ("Immunoglobulin Light-chain Amyloidosis"[MeSH Terms] OR "Primary Systemic Amyloidosis"[MeSH Terms] OR "AL Amyloidosis"[MeSH Terms] OR "Amyloidosis, Primary"[MeSH Terms] OR "Amyloidosis, Immunoglobulin Light-chain"[MeSH Terms] OR "Amyloidoses, Primary"[MeSH Terms] OR "Primary Amyloidoses"[MeSH Terms] OR "AL Amyloidoses"[MeSH Terms] OR "Amyloidoses, Primary Systemic"[MeSH Terms] OR "Amyloidosis, Immunoglobulin Light chain"[MeSH Terms] OR "Amyloidosis, Primary Systemic"[MeSH Terms] OR "Immunoglobulin Light chain Amyloidosis"[MeSH Terms] OR "Immunoglobulin Light-chain Amyloidoses"[MeSH Terms] OR "Primary Systemic Amyloidoses"[MeSH Terms] OR "Systemic Amyloidoses, Primary"[MeSH Terms] OR "Systemic Amyloidosis, Primary"[MeSH Terms]) |

**Table 4. Literature search strategy of Bladder cancer**

| Disease area | BLADDER CANCER |
| --- | --- |
| Search date | **2022.12.06.** |
| Search terms | ("bladder ca"[Title/Abstract] OR "Bladder Cancer"[Title/Abstract] OR "bladder cancer (diagnosis)"[Title/Abstract] OR "BLADDER CANCER MALIGNANT"[Title/Abstract] OR "Bladder cancer NOS"[Title/Abstract] OR "Bladder Cancers"[Title/Abstract] OR "Bladder neoplasms malignant"[Title/Abstract] OR "Bladder--Cancer"[Title/Abstract] OR "CA - Bladder cancer"[Title/Abstract] OR "Cancer of Bladder"[Title/Abstract] OR "Cancer of the Bladder"[Title/Abstract] OR "Cancer, Bladder"[Title/Abstract] OR "Cancer, Urinary Bladder"[Title/Abstract] OR "Malignant Bladder Neoplasm"[Title/Abstract] OR "Malignant Bladder Tumor"[Title/Abstract] OR "Malignant neoplasm of bladder"[Title/Abstract] OR "malignant neoplasm of bladder (diagnosis)"[Title/Abstract] OR "Malignant neoplasm of bladder, NOS"[Title/Abstract] OR "Malignant neoplasm of bladder, part unspecified"[Title/Abstract] OR "Malignant neoplasm of bladder, unspecified"[Title/Abstract] OR "Malignant Neoplasm of the Bladder"[Title/Abstract] OR "Malignant Neoplasm of the Urinary Bladder"[Title/Abstract] OR "Malignant neoplasm of urinary bladder"[Title/Abstract] OR "Malignant Neoplasm, Bladder"[Title/Abstract] OR "Malignant Neoplasm, Urinary Bladder"[Title/Abstract] OR "malignant neosplasm of the bladder"[Title/Abstract] OR "malignant tumor of bladder"[Title/Abstract] OR "Malignant Tumor of the Bladder"[Title/Abstract] OR "Malignant Tumor of the Urinary Bladder"[Title/Abstract] OR "Malignant Tumor of Urinary Bladder"[Title/Abstract] OR "Malignant tumor of urinary bladder (disorder)"[Title/Abstract] OR "Malignant Tumor, Urinary Bladder"[Title/Abstract] OR "Malignant tumour of urinary bladder"[Title/Abstract] OR "Malignant Urinary Bladder Neoplasm"[Title/Abstract] OR "Malignant Urinary Bladder Tumor"[Title/Abstract] OR "Neoplasm malig;bladder"[Title/Abstract] OR "Urinary Bladder Cancer"[Title/Abstract] OR "Urinary Bladder Malignant Neoplasm"[Title/Abstract] OR "Urinary Bladder Malignant Tumor"[Title/Abstract]) OR ("bladder ca"[MeSH Terms] OR "Bladder Cancer"[MeSH Terms] OR "bladder cancer (diagnosis)"[MeSH Terms] OR "BLADDER CANCER MALIGNANT"[MeSH Terms] OR "Bladder cancer NOS"[MeSH Terms] OR "Bladder Cancers"[MeSH Terms] OR "Bladder neoplasms malignant"[MeSH Terms] OR "Bladder--Cancer"[MeSH Terms] OR "CA - Bladder cancer"[MeSH Terms]) |

**Table 5. Literature search strategy of Brain tumor**

| Disease area | BRAIN TUMOR |
| --- | --- |
| Search date | **2022.12.06.** |
| Search terms | ("Brain Neoplasms"[Title/Abstract] OR "Brain Tumors"[Title/Abstract] OR "Neoplasms, Brain"[Title/Abstract] OR "Neoplasm, Brain"[Title/Abstract] OR "Brain Neoplasm"[Title/Abstract] OR "Brain Tumor"[Title/Abstract] OR "Tumor, Brain"[Title/Abstract] OR "Neoplasm of brain"[Title/Abstract] OR "Neoplasm of brain (disorder)"[Title/Abstract] OR "Brain tumour"[Title/Abstract] OR "Neoplasm of unspecified nature of brain"[Title/Abstract] OR "Brain neoplasm NOS"[Title/Abstract] OR "Brain tumor NOS"[Title/Abstract] OR "Brain tumour NOS"[Title/Abstract] OR "neoplasm of brain (diagnosis)"[Title/Abstract] OR "brain tumor (diagnosis)"[Title/Abstract] OR "Neoplasm of the Brain"[Title/Abstract] OR "Tumor of Brain"[Title/Abstract] OR "Tumor of the Brain"[Title/Abstract] OR "brain cancer"[Title/Abstract] OR "brain tumours"[Title/Abstract] OR "Brain--Tumors"[Title/Abstract] OR "BT - Brain tumour"[Title/Abstract] OR "BT - Brain tumor"[Title/Abstract] OR ""[Title/Abstract]) OR ("Brain Neoplasms"[MeSH Terms] OR "Brain Tumors"[MeSH Terms] OR "Neoplasms, Brain"[MeSH Terms] OR "Neoplasm, Brain"[MeSH Terms] OR "Brain Neoplasm"[MeSH Terms] OR "Brain Tumor"[MeSH Terms] OR "Tumor, Brain"[MeSH Terms]) |

**Table 6. Literature search strategy of Breast cancer**

| Disease area | BREAST CANCER |
| --- | --- |
| Search date | **2022.09.26.** |
| Search terms | ("breast ca"[Title/Abstract] OR "breast cancer"[Title/Abstract] OR "breast cancer (diagnosis)"[Title/Abstract] OR "breast cancer nos"[Title/Abstract] OR "breast cancer stage unspecified"[Title/Abstract] OR "breast cancers"[Title/Abstract] OR "breast malignant neoplasm"[Title/Abstract] OR "breast malignant neoplasms"[Title/Abstract] OR "breast malignant tumor"[Title/Abstract] OR "breast malignant tumors"[Title/Abstract] OR "breast tumor malignant"[Title/Abstract] OR "breast tumour malignant"[Title/Abstract] OR "breast--cancer"[Title/Abstract] OR "ca - breast cancer"[Title/Abstract] OR "cancer of breast"[Title/Abstract] OR "cancer of the breast"[Title/Abstract] OR "cancer, breast"[Title/Abstract] OR "cancer, mammary"[Title/Abstract] OR "cancers, mammary"[Title/Abstract] OR "malignant breast neoplasm"[Title/Abstract] OR "malignant breast tumor"[Title/Abstract] OR "malignant neoplasm breast"[Title/Abstract] OR "malignant neoplasm of breast"[Title/Abstract] OR "malignant neoplasm of breast (diagnosis)"[Title/Abstract] OR "malignant neoplasm of breast (disorder)"[Title/Abstract] OR "malignant neoplasm of breast, unspecified"[Title/Abstract] OR "malignant neoplasm of breast, unspecified part"[Title/Abstract] OR "malignant neoplasm of the breast"[Title/Abstract] OR "malignant neoplasms of breast (c50)"[Title/Abstract] OR "malignant tumor of breast"[Title/Abstract] OR "malignant tumor of the breast"[Title/Abstract] OR "malignant tumour of breast"[Title/Abstract] OR "mammary cancer"[Title/Abstract] OR "mammary cancers"[Title/Abstract] OR ""[Title/Abstract]) OR (Breast Cancer[MeSH Terms] OR Malignant Tumor of Breast[MeSH Terms] OR Mammary Cancer[MeSH Terms] OR Malignant Neoplasm of Breast[MeSH Terms] OR Cancer of Breast[MeSH Terms] OR Cancer of the Breast[MeSH Terms] OR Breast Malignant Neoplasms[MeSH Terms] OR Breast Malignant Neoplasm[MeSH Terms] OR Cancer, Breast[MeSH Terms] OR Breast Malignant Tumors[MeSH Terms] OR Breast Malignant Tumor[MeSH Terms] OR Cancer, Mammary[MeSH Terms] OR Cancers, Mammary[MeSH Terms] OR Mammary Cancers[MeSH Terms]) |

**Table 7. Literature search strategy of Chronic lymphocytic leukaemia**

| Disease area | CHRONIC LYMPHOCYTIC LEUKAEMIA |
| --- | --- |
| Search date | **2022.12.06.** |
| Search terms | "Lymphomas, Lymphocytic"[MeSH Terms] OR "Lymphomas, Small Lymphocytic"[MeSH Terms] OR "Lymphomas, Small-Cell"[MeSH Terms] OR "Lymphomas, Well-Differentiated Lymphocytic"[MeSH Terms] OR "Lymphoplasmacytoid Lymphomas, CLL"[MeSH Terms] OR "Small Cell Lymphoma"[MeSH Terms] OR "Small Lymphocytic Lymphoma"[MeSH Terms] OR "Small Lymphocytic Lymphomas"[MeSH Terms] OR "Small-Cell Lymphomas"[MeSH Terms] OR "Well-Differentiated Lymphocytic Lymphoma"[MeSH Terms] OR "Well-Differentiated Lymphocytic Lymphomas"[MeSH Terms] OR "Diffuse Well Differentiated Lymphocytic Lymphoma"[MeSH Terms] OR "Chronic Lymphatic Leukemia"[MeSH Terms] OR "B Cell Chronic Lymphocytic Leukemia"[MeSH Terms] OR "B Cell Malignancy, Low Grade"[MeSH Terms] OR "B-Cell Malignancies, Low-Grade"[MeSH Terms] OR "Chronic Lymphatic Leukemias"[MeSH Terms] OR "Disrupted In B Cell Malignancy"[MeSH Terms] OR "Leukemias, Chronic Lymphatic"[MeSH Terms] OR "Low-Grade B-Cell Malignancies"[MeSH Terms] OR "Low-Grade B-Cell Malignancy"[MeSH Terms] OR "Lymphatic Leukemia, Chronic"[MeSH Terms] OR "Lymphatic Leukemias, Chronic"[MeSH Terms] OR "Malignancies, Low-Grade B-Cell"[MeSH Terms] OR "Malignancy, Low-Grade B-Cell"[MeSH Terms]) |

**Table 8. Literature search strategy of Chronic myeloid leukaemia**

| Disease area | CHRONIC MYELOID LEUKAEMIA |
| --- | --- |
| Search date | **2022.12.06.** |
| Search terms | ("Myeloid Leukemia, Chronic"[Title/Abstract] OR "Chronic myeloid leukemia"[Title/Abstract] OR "Chronic myeloid leukemia, disease (disorder)"[Title/Abstract] OR "Chronic myeloid leukemia (morphologic abnormality)"[Title/Abstract] OR "Chronic granulocytic leukemia"[Title/Abstract] OR "Chronic myelogenous leukemia"[Title/Abstract] OR "CGL - Chronic granulocytic leukemia"[Title/Abstract] OR "CML - Chronic myeloid leukemia"[Title/Abstract] OR "Chronic myelocytic leukemia"[Title/Abstract] OR "Chronic myeloid leukemia, disease"[Title/Abstract] OR "Chronic myeloid leukaemia"[Title/Abstract] OR "CGL - Chronic granulocytic leukaemia"[Title/Abstract] OR "CML - Chronic myeloid leukaemia"[Title/Abstract] OR "Chronic granulocytic leukaemia"[Title/Abstract] OR "Chronic myelocytic leukaemia"[Title/Abstract] OR "Chronic myelogenous leukaemia"[Title/Abstract] OR "Chronic myeloid leukaemia, disease"[Title/Abstract] OR "Leukaemias chronic myeloid"[Title/Abstract] OR "Leukemias chronic myeloid"[Title/Abstract] OR "CML"[Title/Abstract] OR "Myeloid leukaemia, chronic"[Title/Abstract] OR "Leukemia myelocytic chronic"[Title/Abstract] OR "Leukaemia myelocytic chronic"[Title/Abstract] OR "LEUKEMIA, CHRONIC MYELOID"[Title/Abstract] OR "LEUKEMIA, CHRONIC MYELOGENOUS"[Title/Abstract] OR "chronic myelogenous leukemia (diagnosis)"[Title/Abstract] OR "chronic myelogenous leukemia (CML)"[Title/Abstract] OR "CML (chronic myelogenous leukemia)"[Title/Abstract] OR "CGL"[Title/Abstract] OR "Chronic Myelogenous Leukemias"[Title/Abstract] OR "granulocytic leukemia, chronic"[Title/Abstract] OR "myelocytic leukemia, chronic"[Title/Abstract] OR "myelogenous leukemia, chronic"[Title/Abstract] OR "CML - Chronic Myelogenous Leukemia"[Title/Abstract] OR "chronic myeloid leukemias"[Title/Abstract] OR "Leukemia, Myeloid, Chronic"[Title/Abstract] OR "chronic; myelosis"[Title/Abstract] OR "leukemia; myeloid, chronic"[Title/Abstract] OR "myeloid; leukemia, chronic"[Title/Abstract] OR "granulocytic; leukemia, chronic"[Title/Abstract] OR "leukemia; granulocytic, chronic"[Title/Abstract] OR "leukemia; myelocytic, chronic"[Title/Abstract] OR "myelocytic; leukemia, chronic"[Title/Abstract] OR "myelosis; chronic"[Title/Abstract] OR "Leukaemia;chronic myeloid"[Title/Abstract] OR "Myelosis"[Title/Abstract] OR "Leukemia, myelomonocytic (Naegeli)"[Title/Abstract] OR "Chronic myelosis"[Title/Abstract] OR "LEUKEMIA, MYELOCYTIC, CHRONIC"[Title/Abstract] OR "LEUKEMIA, GRANULOCYTIC, CHRONIC"[Title/Abstract] OR "LEUKEMIA, MYELOGENOUS, CHRONIC"[Title/Abstract] OR "LEUKEMIA CHRONIC MYELOGENOUS"[Title/Abstract] OR "LEUKEMIA CHRONIC MYELOCYTIC"[Title/Abstract]) OR ("Chronic myeloid leukemia"[MeSH Terms] OR "Chronic myelogenous leukemia"[MeSH Terms] OR "Chronic myelocytic leukemia"[MeSH Terms] OR "Chronic Myelogenous Leukemias"[MeSH Terms]) |

**Table 9. Literature search strategy of Digestive cancer**

| Disease area | DIGESTIVE CANCER |
| --- | --- |
| Search date | **2022.12.07.** |
| Search terms | ("Malignant neoplasm of gastrointestinal tract"[Title/Abstract] OR "Cancer of Gastrointestinal Tract"[Title/Abstract] OR "Gastrointestinal Cancer"[Title/Abstract] OR "Cancer of the Gastrointestinal Tract"[Title/Abstract] OR "Cancer, Gastrointestinal"[Title/Abstract] OR "Cancers, Gastrointestinal"[Title/Abstract] OR "Gastrointestinal Cancers"[Title/Abstract] OR "Gastrointestinal Tract Cancers"[Title/Abstract] OR "Gastrointestinal Tract Cancer"[Title/Abstract] OR "Malignant neoplasm of gastrointestinal tract (disorder)"[Title/Abstract] OR "Malignant gastrointestinal tract tumors"[Title/Abstract] OR "Malignant GI tract tumors"[Title/Abstract] OR "Malignant gastrointestinal tract tumours"[Title/Abstract] OR "Malignant GI tract tumours"[Title/Abstract] OR "Gastrointestinal neoplasm malignant"[Title/Abstract] OR "GI neoplasm malignant"[Title/Abstract] OR "Gastrointestinal tract cancer NOS"[Title/Abstract] OR "gastrointestinal cancer (diagnosis)"[Title/Abstract] OR "malignant neoplasm of gastrointestinal tract (diagnosis)"[Title/Abstract] OR "GI cancer"[Title/Abstract] OR "malignant neoplasm of GI tract"[Title/Abstract] OR "malignant tumor of gastrointestinal tract"[Title/Abstract] OR "Malignant Digestive System Neoplasm"[Title/Abstract] OR "Gastrointestinal System Cancer"[Title/Abstract] OR "Malignant Gastrointestinal System Neoplasm"[Title/Abstract] OR "Malignant Gastrointestinal Neoplasm"[Title/Abstract] OR "digestive cancer"[Title/Abstract] OR "cancer digestive"[Title/Abstract] OR "cancer gastrointestinal"[Title/Abstract] OR "cancer gi"[Title/Abstract] OR "cancers digestive"[Title/Abstract] OR "cancers gastrointestinal tract"[Title/Abstract] OR "cancers gi"[Title/Abstract] OR "digestive cancers"[Title/Abstract] OR "Malignant neoplasm of gastrointestinal tract NOS"[Title/Abstract] OR "Gastrointestinal system--Cancer"[Title/Abstract] OR "Malignant neoplasm of gastrointestinal tract, NOS"[Title/Abstract]) OR ("Cancer of Gastrointestinal Tract"[MeSH Terms] OR "Gastrointestinal Cancer"[MeSH Terms] OR "Cancer of the Gastrointestinal Tract"[MeSH Terms] OR "Cancer, Gastrointestinal"[MeSH Terms] OR "Cancers, Gastrointestinal"[MeSH Terms] OR "Gastrointestinal Cancers"[MeSH Terms] OR "Gastrointestinal Tract Cancers"[MeSH Terms] OR "Gastrointestinal Tract Cancer"[MeSH Terms]) |

**Table 10. Literature search strategy of Haemochromatosis**

| Disease area | HAEMOCHROMATOSIS |
| --- | --- |
| Search date | **2022.12.07.** |
| Search terms | ("Hemochromatosis"[Title/Abstract] OR "Diabetes, Bronze"[Title/Abstract] OR "Bronzed Cirrhosis"[Title/Abstract] OR "Iron Storage Disorder"[Title/Abstract] OR "Pigmentary Cirrhosis"[Title/Abstract] OR "Troisier-Hanot-Chauffard Syndrome"[Title/Abstract] OR "Von Recklenhausen-Applebaum Disease"[Title/Abstract] OR "Hemochromatoses"[Title/Abstract] OR "Haemochromatosis"[Title/Abstract] OR "Bronze Diabetes"[Title/Abstract] OR "Hemochromatose"[Title/Abstract] OR "Haemochromatoses"[Title/Abstract] OR "Syndrome, Troisier-Hanot-Chauffard"[Title/Abstract] OR "Bronzed Cirrhoses"[Title/Abstract] OR "Cirrhoses, Bronzed"[Title/Abstract] OR "Cirrhoses, Pigmentary"[Title/Abstract] OR "Cirrhosis, Bronzed"[Title/Abstract] OR "Cirrhosis, Pigmentary"[Title/Abstract] OR "Disease, Von Recklenhausen-Applebaum"[Title/Abstract] OR "Diseases, Von Recklenhausen-Applebaum"[Title/Abstract] OR "Disorder, Iron Storage"[Title/Abstract] OR "Disorders, Iron Storage"[Title/Abstract] OR "Iron Storage Disorders"[Title/Abstract] OR "Pigmentary Cirrhoses"[Title/Abstract] OR "Recklenhausen-Applebaum Disease, Von"[Title/Abstract] OR "Recklenhausen-Applebaum Diseases, Von"[Title/Abstract] OR "Storage Disorder, Iron"[Title/Abstract] OR "Storage Disorders, Iron"[Title/Abstract] OR "Syndromes, Troisier-Hanot-Chauffard"[Title/Abstract] OR "Troisier Hanot Chauffard Syndrome"[Title/Abstract] OR "Troisier-Hanot-Chauffard Syndromes"[Title/Abstract] OR "Von Recklenhausen Applebaum Disease"[Title/Abstract] OR "Von Recklenhausen-Applebaum Diseases"[Title/Abstract] OR "Bronze diabetes (disorder)"[Title/Abstract] OR "Hemochromatosis (disorder)"[Title/Abstract] OR "Iron storage disease"[Title/Abstract] OR "Bronzed diabetes"[Title/Abstract] OR "Hematochromatosis"[Title/Abstract] OR "hemochromatosis (diagnosis)"[Title/Abstract] OR "bronze diabetes (diagnosis)"[Title/Abstract] OR "Hemosiderosis"[Title/Abstract] OR "diabetes bronze"[Title/Abstract] OR "disorders iron storage"[Title/Abstract] OR "Iron Overload Disease"[Title/Abstract] OR "Hanot-Chauffard"[Title/Abstract] OR "bronzed; diabetes"[Title/Abstract] OR "diabetes; bronzed"[Title/Abstract] OR "iron; storage disorder"[Title/Abstract] OR "Hemochromatosis, unspecified"[Title/Abstract] OR "Rare hereditary hemochromatosis"[Title/Abstract] OR "Hemochromatosis NOS"[Title/Abstract] OR "Hemochromatosis, NOS"[Title/Abstract] OR "von Recklinghausen-Appelbaum disease"[Title/Abstract] OR "Iron storage disease, NOS"[Title/Abstract] OR "iron accumulation disorders"[Title/Abstract]) OR ("Hemochromatosis"[MeSH Terms] OR "Diabetes, Bronze"[MeSH Terms] OR "Bronzed Cirrhosis"[MeSH Terms] OR "Iron Storage Disorder"[MeSH Terms] OR "Pigmentary Cirrhosis"[MeSH Terms] OR "Troisier-Hanot-Chauffard Syndrome"[MeSH Terms] OR "Von Recklenhausen-Applebaum Disease"[MeSH Terms] OR "Hemochromatoses"[MeSH Terms] OR "Haemochromatosis"[MeSH Terms] OR "Bronze Diabetes"[MeSH Terms] OR "Hemochromatose"[MeSH Terms] OR "Haemochromatoses"[MeSH Terms] OR "Syndrome, Troisier-Hanot-Chauffard"[MeSH Terms] OR "Bronzed Cirrhoses"[MeSH Terms] OR "Cirrhoses, Bronzed"[MeSH Terms] OR "Cirrhoses, Pigmentary"[MeSH Terms] OR "Cirrhosis, Bronzed"[MeSH Terms] OR "Cirrhosis, Pigmentary"[MeSH Terms] OR "Disease, Von Recklenhausen-Applebaum"[MeSH Terms] OR "Diseases, Von Recklenhausen-Applebaum"[MeSH Terms] OR "Disorder, Iron Storage"[MeSH Terms] OR "Disorders, Iron Storage"[MeSH Terms] OR "Iron Storage Disorders"[MeSH Terms] OR "Pigmentary Cirrhoses"[MeSH Terms] OR "Recklenhausen-Applebaum Disease, Von"[MeSH Terms] OR "Recklenhausen-Applebaum Diseases, Von"[MeSH Terms] OR "Storage Disorder, Iron"[MeSH Terms] OR "Storage Disorders, Iron"[MeSH Terms] OR "Syndromes, Troisier-Hanot-Chauffard"[MeSH Terms] OR "Troisier Hanot Chauffard Syndrome"[MeSH Terms] OR "Troisier-Hanot-Chauffard Syndromes"[MeSH Terms] OR "Von Recklenhausen Applebaum Disease"[MeSH Terms] OR "Von Recklenhausen-Applebaum Diseases"[MeSH Terms]) |

**Table 11. Literature search strategy of Haemophilia**

| Disease area | HAEMOPHILIA |
| --- | --- |
| Search date | **2022.12.07.** |
| Search terms | ("Hemophilia, NOS"[Title/Abstract] OR "Hemophilia"[Title/Abstract] OR "Hemophilia (disorder)"[Title/Abstract] OR "Haemophilia"[Title/Abstract] OR "Haemophilia NOS"[Title/Abstract] OR "Hemophilia NOS"[Title/Abstract] OR "hemophilias"[Title/Abstract] OR "hemophilia disorder"[Title/Abstract] OR "Haemophilia, NOS"[Title/Abstract]) OR ("Hemophilia, NOS"[MeSH Terms] OR "Hemophilia"[MeSH Terms] OR "Hemophilia (disorder)"[MeSH Terms] OR "Haemophilia"[MeSH Terms] OR "Haemophilia NOS"[MeSH Terms] OR "Hemophilia NOS"[MeSH Terms] OR "hemophilias"[MeSH Terms] OR "hemophilia disorder"[MeSH Terms] OR "Haemophilia, NOS"[MeSH Terms]) |

**Table 12. Literature search strategy of Idiopathic thrombocytopenic purpura**

| Disease area | IDIOPATHIC THROMBOCYTOPENIC PURPURA |
| --- | --- |
| Search date | **2022.12.07.** |
| Search terms | ("idiopathic thrombocytopenic purpura"[Title/Abstract] OR "idiopathic thrombocytopenic purpuras"[Title/Abstract] OR "purpura, idiopathic thrombocytopenic"[Title/Abstract] OR "purpuras, idiopathic thrombocytopenic"[Title/Abstract] OR "idiopathic thrombocytopenic purpura (disorder)"[Title/Abstract] OR "idiopathic thrombocytopenic purpura (diagnosis)"[Title/Abstract] OR "idiopathic thrombocytopenic purpura, NOS"[Title/Abstract]) OR ("idiopathic thrombocytopenic purpura"[MeSH Terms] OR "idiopathic thrombocytopenic purpuras"[MeSH Terms] OR "purpura, idiopathic thrombocytopenic"[MeSH Terms] OR "purpuras, idiopathic thrombocytopenic"[MeSH Terms]) |

**Table 13. Literature search strategy of Kidney cancer**

| Disease area | KIDNEY CANCER |
| --- | --- |
| Search date | **2022.12.07.** |
| Search terms | ("Malignant neoplasm of kidney"[Title/Abstract] OR "Cancer of Kidney"[Title/Abstract] OR "Renal Cancer"[Title/Abstract] OR "Cancer of the Kidney"[Title/Abstract] OR "Kidney Cancer"[Title/Abstract] OR "Cancers, Kidney"[Title/Abstract] OR "Kidney Cancers"[Title/Abstract] OR "Cancer, Renal"[Title/Abstract] OR "Cancers, Renal"[Title/Abstract] OR "Renal Cancers"[Title/Abstract] OR "Cancer, Kidney"[Title/Abstract] OR "Malignant tumor of kidney"[Title/Abstract] OR "Malignant tumor of kidney (disorder)"[Title/Abstract] OR "Renal malignant tumor"[Title/Abstract] OR "CA - Cancer of kidney"[Title/Abstract] OR "CA - Renal cancer"[Title/Abstract] OR "Malignant tumour of kidney"[Title/Abstract] OR "Renal malignant tumour"[Title/Abstract] OR "Renal neoplasms malignant"[Title/Abstract] OR "Malignant renal neoplasm"[Title/Abstract] OR "malignant neoplasm of kidney (diagnosis)"[Title/Abstract] OR "kidney cancer (diagnosis)"[Title/Abstract] OR "Malignant Kidney Neoplasm"[Title/Abstract] OR "Malignant Neoplasm of the Kidney"[Title/Abstract] OR "Malignant Kidney Tumor"[Title/Abstract] OR "Malignant Renal Tumor"[Title/Abstract] OR "Malignant Tumor of the Kidney"[Title/Abstract] OR "renal cell cancer"[Title/Abstract] OR "Kidney (renal cell) cancer"[Title/Abstract] OR "malignant renal tumors"[Title/Abstract] OR "malignant neosplasm of the kidney"[Title/Abstract] OR "Neoplasm malig;kidney"[Title/Abstract] OR "Malignant neoplasm of kidney NOS"[Title/Abstract] OR "Kidneys--Cancer"[Title/Abstract] OR "Malignant neoplasm of kidney, NOS"[Title/Abstract] OR "Renal malignant neoplasm"[Title/Abstract] OR ""[Title/Abstract]) OR ("Cancer of Kidney"[MeSH Terms] OR "Renal Cancer"[MeSH Terms] OR "Cancer of the Kidney"[MeSH Terms] OR "Kidney Cancer"[MeSH Terms] OR "Cancers, Kidney"[MeSH Terms] OR "Kidney Cancers"[MeSH Terms] OR "Cancer, Renal"[MeSH Terms] OR "Cancers, Renal"[MeSH Terms] OR "Renal Cancers"[MeSH Terms] OR "Cancer, Kidney"[MeSH Terms]) |

**Table 14. Literature search strategy of Lung cancer**

| Disease area | LUNG CANCER |
| --- | --- |
| Search date | **2022.12.07.** |
| Search terms | ("Malignant neoplasm of lung"[Title/Abstract] OR "Lung Cancer"[Title/Abstract] OR "Pulmonary Cancer"[Title/Abstract] OR "Cancer of Lung"[Title/Abstract] OR "Cancer of the Lung"[Title/Abstract] OR "Cancer, Lung"[Title/Abstract] OR "Cancers, Lung"[Title/Abstract] OR "Lung Cancers"[Title/Abstract] OR "Cancer, Pulmonary"[Title/Abstract] OR "Cancers, Pulmonary"[Title/Abstract] OR "Pulmonary Cancers"[Title/Abstract] OR "Malignant tumor of lung"[Title/Abstract] OR "Malignant tumor of lung (disorder)"[Title/Abstract] OR "CA - Lung cancer"[Title/Abstract] OR "Malignant tumour of lung"[Title/Abstract] OR "Lung neoplasm malignant"[Title/Abstract] OR "Lung cancer NOS"[Title/Abstract] OR "Malignant lung tumors"[Title/Abstract] OR "lung cancer (diagnosis)"[Title/Abstract] OR "malignant neoplasm of lung (diagnosis)"[Title/Abstract] OR "malignant lung neoplasm"[Title/Abstract] OR "Malignant Neoplasm of the Lung"[Title/Abstract] OR "Malignant Lung Tumor"[Title/Abstract] OR "Malignant Tumor of the Lung"[Title/Abstract] OR "lung malignancies"[Title/Abstract] OR "malignant neoplasm lung"[Title/Abstract] OR "cancer pulmonary"[Title/Abstract] OR "cancers lungs"[Title/Abstract] OR "lung malignancy"[Title/Abstract] OR "lung malignant tumors"[Title/Abstract] OR "lung malignant tumours"[Title/Abstract] OR "lungs cancer"[Title/Abstract] OR "malignant neosplasm of the lung"[Title/Abstract] OR "Neoplasm malig;lung"[Title/Abstract] OR "Lungs--Cancer"[Title/Abstract] OR "Malignant neoplasm of lung, NOS"[Title/Abstract] OR "LUNG CANCER MALIGNANT"[Title/Abstract]) OR ("Lung Cancer"[MeSH Terms] OR "Pulmonary Cancer"[MeSH Terms] OR "Cancer of Lung"[MeSH Terms] OR "Cancer of the Lung"[MeSH Terms] OR "Cancer, Lung"[MeSH Terms] OR "Cancers, Lung"[MeSH Terms] OR "Lung Cancers"[MeSH Terms] OR "Cancer, Pulmonary"[MeSH Terms] OR "Cancers, Pulmonary"[MeSH Terms] OR "Pulmonary Cancers"[MeSH Terms]) |

**Table 15. Literature search strategy of Lymphomas**

| Disease area | LYMPHOMAS |
| --- | --- |
| Search date | **2022.12.07.** |
| Search terms | ("Lymphoma"[Title/Abstract] OR "Germinoblastoma"[Title/Abstract] OR "Reticulolymphosarcoma"[Title/Abstract] OR "Sarcoma, Germinoblastic"[Title/Abstract] OR "Lymphoma, Malignant"[Title/Abstract] OR "Lymphomas"[Title/Abstract] OR "Germinoblastic Sarcomas"[Title/Abstract] OR "Germinoblastomas"[Title/Abstract] OR "Reticulolymphosarcomas"[Title/Abstract] OR "Sarcomas, Germinoblastic"[Title/Abstract] OR "Lymphomas, Malignant"[Title/Abstract] OR "Malignant Lymphomas"[Title/Abstract] OR "Germinoblastic Sarcoma"[Title/Abstract] OR "Malignant Lymphoma"[Title/Abstract] OR "Malignant lymphoma (disorder)"[Title/Abstract] OR "Malignant lymphoma (morphologic abnormality)"[Title/Abstract] OR "Lymphosarcoma"[Title/Abstract] OR "Cancer of lymphatic system"[Title/Abstract] OR "Lymphoma NOS"[Title/Abstract] OR "Lymphoma malignant"[Title/Abstract] OR "Malignant lymphoma NOS"[Title/Abstract] OR "malignant lymphoma (diagnosis)"[Title/Abstract] OR "Lymphoma (Hodgkin's and Non-Hodgkin's)"[Title/Abstract] OR "Lymphoma (Hodgkin and Non-Hodgkin)"[Title/Abstract] OR "Lymphomatous"[Title/Abstract] OR "lymphomas malignant"[Title/Abstract] OR "Malignant lymphoma, NOS"[Title/Abstract] OR "Lymphoma, NOS"[Title/Abstract] OR "Lymphoma morphology"[Title/Abstract]) OR ("Lymphoma"[MeSH Terms] OR "Germinoblastoma"[MeSH Terms] OR "Reticulolymphosarcoma"[MeSH Terms] OR "Sarcoma, Germinoblastic"[MeSH Terms] OR "Lymphoma, Malignant"[MeSH Terms] OR "Lymphomas"[MeSH Terms] OR "Germinoblastic Sarcomas"[MeSH Terms] OR "Germinoblastomas"[MeSH Terms] OR "Reticulolymphosarcomas"[MeSH Terms] OR "Sarcomas, Germinoblastic"[MeSH Terms] OR "Lymphomas, Malignant"[MeSH Terms] OR "Malignant Lymphomas"[MeSH Terms] OR "Germinoblastic Sarcoma"[MeSH Terms] OR "Malignant Lymphoma"[MeSH Terms]) |

**Table 16. Literature search strategy of Melanoma**

| Disease area | MELANOMA |
| --- | --- |
| Search date | **2022.12.07.** |
| Search terms | ("melanoma"[Title/Abstract] OR "MALIGNANT MELANOMA"[Title/Abstract] OR "Melanomas"[Title/Abstract] OR "Malignant Melanomas"[Title/Abstract] OR "Melanoma, Malignant"[Title/Abstract] OR "Melanomas, Malignant"[Title/Abstract] OR "Malignant melanoma (disorder)"[Title/Abstract] OR "Malignant melanoma (morphologic abnormality)"[Title/Abstract] OR "Melanosarcoma"[Title/Abstract] OR "MM - malignant melanoma"[Title/Abstract] OR "Cancer of skin pigment cells"[Title/Abstract] OR "Melanoma malignant"[Title/Abstract] OR "Malignant melanoma NOS"[Title/Abstract] OR "malignant melanoma (diagnosis)"[Title/Abstract] OR "malignant neoplasm melanoma"[Title/Abstract] OR "cutaneous melanoma"[Title/Abstract] OR "melanocarcinoma"[Title/Abstract] OR "melanoma syndrome"[Title/Abstract] OR "nevocarcinoma"[Title/Abstract] OR "Malignant melanoma, NOS"[Title/Abstract] OR "Melanoma, NOS"[Title/Abstract]) OR (""[MeSH Terms]) |

**Table 17. Literature search strategy of Myelodysplastic syndromes**

| Disease area | MYELODYSPLASTIC SYNDROMES |
| --- | --- |
| Search date | **2022.12.15.** |
| Search terms | ("MYELODYSPLASTIC SYNDROME"[Title/Abstract] OR "Myelodysplastic Syndromes"[Title/Abstract] OR "Dysmyelopoietic Syndromes"[Title/Abstract] OR "Syndromes, Myelodysplastic"[Title/Abstract] OR "Myelodysplastic Syndrome"[Title/Abstract] OR "Syndrome, Myelodysplastic"[Title/Abstract] OR "Dysmyelopoietic Syndrome"[Title/Abstract] OR "Syndrome, Dysmyelopoietic"[Title/Abstract] OR "Syndromes, Dysmyelopoietic"[Title/Abstract] OR "Myelodysplastic syndrome (clinical)"[Title/Abstract] OR "Myelodysplastic syndrome (disorder)"[Title/Abstract] OR "Myelodysplastic syndrome (morphologic abnormality)"[Title/Abstract] OR "MDS - Myelodysplastic syndrome"[Title/Abstract] OR "Smoldering leukemia"[Title/Abstract] OR "Smouldering leukaemia"[Title/Abstract] OR "Myelodysplasia"[Title/Abstract] OR "Myeloid dysplasia"[Title/Abstract] OR "Myelodysplastic syndrome NOS"[Title/Abstract] OR "Myelodysplastic syndrome, unspecified"[Title/Abstract] OR "Dysmyelopoiesis"[Title/Abstract] OR "Myelodysplastic syndrome (MDS)"[Title/Abstract] OR "MYELODYSPLASTIC SYNDROME, SUSCEPTIBILITY TO"[Title/Abstract] OR OR "myelodysplastic syndrome (diagnosis)"[Title/Abstract] OR "Oligoblastic Leukemia"[Title/Abstract] OR "Hematopoeitic - Myelodysplastic Syndrome (MDS)"[Title/Abstract] OR "Myelodysplastic Neoplasm"[Title/Abstract] OR "Myelodysplastic Syndrome/Neoplasm"[Title/Abstract] OR "preleukemia"[Title/Abstract] OR "preleukemic; syndrome"[Title/Abstract] OR "myelodysplastic; syndrome"[Title/Abstract] OR "syndrome; myelodysplastic"[Title/Abstract] OR "syndrome; preleukemic"[Title/Abstract] OR "Myelodysplastic syndrome, NOS"[Title/Abstract] OR "Preleukemic syndrome"[Title/Abstract] OR "Preleukaemia"[Title/Abstract] OR "Preleukaemic syndrome"[Title/Abstract]) OR ("Myelodysplastic Syndromes"[MeSH Terms] OR "Dysmyelopoietic Syndromes"[MeSH Terms] OR "Syndromes, Myelodysplastic"[MeSH Terms] OR "Myelodysplastic Syndrome"[MeSH Terms] OR "Syndrome, Myelodysplastic"[MeSH Terms] OR "Dysmyelopoietic Syndrome"[MeSH Terms] OR "Syndrome, Dysmyelopoietic"[MeSH Terms] OR "Syndromes, Dysmyelopoietic"[MeSH Terms]) |

**Table18. Literature search strategy of Myeloma**

| Disease area | MYELOMA |
| --- | --- |
| Search date | **2022.12.** |
| Search terms | ("Multiple Myeloma"[Title/Abstract] OR "Myelomatosis"[Title/Abstract] OR "Myeloma, Plasma-Cell"[Title/Abstract] OR "Kahler Disease"[Title/Abstract] OR "Myeloma, Multiple"[Title/Abstract] OR "Myeloma-Multiple"[Title/Abstract] OR "Plasma Cell Myeloma"[Title/Abstract] OR "Multiple Myelomas"[Title/Abstract] OR "Myelomas, Multiple"[Title/Abstract] OR "Myeloma Multiple"[Title/Abstract] OR "Myeloma-Multiples"[Title/Abstract] OR "Myeloma, Plasma Cell"[Title/Abstract] OR "Myelomas, Plasma Cell"[Title/Abstract] OR "Plasma Cell Myelomas"[Title/Abstract] OR "Disease, Kahler"[Title/Abstract] OR "Myelomas, Plasma-Cell"[Title/Abstract] OR "Plasma-Cell Myeloma"[Title/Abstract] OR "Plasma-Cell Myelomas"[Title/Abstract] OR "Cell Myeloma, Plasma"[Title/Abstract] OR "Cell Myelomas, Plasma"[Title/Abstract] OR "Myelomatoses"[Title/Abstract] OR "Multiple myeloma (disorder)"[Title/Abstract] OR "Plasma cell myeloma (morphologic abnormality)"[Title/Abstract] OR "Plasmacytic myeloma"[Title/Abstract] OR "Myeloma"[Title/Abstract] OR "Multiple myeloma (clinical)"[Title/Abstract] OR "Kahler's disease"[Title/Abstract] OR "Peripheral plasma cell myeloma"[Title/Abstract] OR "Myelomatosis multiple"[Title/Abstract] OR "multiple myeloma (diagnosis)"[Title/Abstract] OR "plasma cell neoplasm"[Title/Abstract] OR "multiple myeloma and other plasma cell neoplasms"[Title/Abstract] OR "neoplasm, plasma cell"[Title/Abstract] OR "plasma cell neoplasms"[Title/Abstract] OR "Multiple myeloma / Plasma cell neoplasm"[Title/Abstract] OR "multiple myeloma (MM)"[Title/Abstract] OR "multiple myelomatosis"[Title/Abstract] OR "myelomas"[Title/Abstract] OR "Kahler"[Title/Abstract] OR "myelomata; multiple"[Title/Abstract] OR "Myeloma;multiple"[Title/Abstract] OR "Medullary plasmacytoma"[Title/Abstract] OR "Multiple myeloma NOS"[Title/Abstract] OR "Myeloma, NOS"[Title/Abstract] OR "KAHLER-BOZZOLO DISEASE"[Title/Abstract] OR "MULTIPLE MYELOMA MYELOMATOSIS"[Title/Abstract] OR ""[Title/Abstract]) OR ("Multiple Myeloma"[MeSH Terms] OR "Myelomatosis"[MeSH Terms] OR "Myeloma, Plasma-Cell"[MeSH Terms] OR "Kahler Disease"[MeSH Terms] OR "Myeloma, Multiple"[MeSH Terms] OR "Myeloma-Multiple"[MeSH Terms] OR "Plasma Cell Myeloma"[MeSH Terms] OR "Multiple Myelomas"[MeSH Terms] OR "Myelomas, Multiple"[MeSH Terms] OR "Myeloma Multiple"[MeSH Terms] OR "Myeloma-Multiples"[MeSH Terms] OR "Myeloma, Plasma Cell"[MeSH Terms] OR "Myelomas, Plasma Cell"[MeSH Terms] OR "Plasma Cell Myelomas"[MeSH Terms] OR "Disease, Kahler"[MeSH Terms] OR "Myelomas, Plasma-Cell"[MeSH Terms] OR "Plasma-Cell Myeloma"[MeSH Terms] OR "Plasma-Cell Myelomas"[MeSH Terms] OR "Cell Myeloma, Plasma"[MeSH Terms] OR "Cell Myelomas, Plasma"[MeSH Terms] OR "Myelomatoses"[MeSH Terms]) |

**Table 19. Literature search strategy of Myeloproliferative neoplasms**

| Disease area | MYELOPROLIFERATIVE NEOPLASMS |
| --- | --- |
| Search date | **2022.12.07.** |
| Search terms | ("Myeloproliferative disease"[Title/Abstract] OR "Myeloproliferative Disorders"[Title/Abstract] OR "Disorder, Myeloproliferative"[Title/Abstract] OR "Disorders, Myeloproliferative"[Title/Abstract] OR "Myeloproliferative Disorder"[Title/Abstract] OR "Proliferation of myeloid cells"[Title/Abstract] OR "Myeloproliferative disorder (morphologic abnormality)"[Title/Abstract] OR "Myeloproliferative disorder (disorder)"[Title/Abstract] OR "Myeloproliferative neoplasm"[Title/Abstract] OR "Disorder myeloproliferative"[Title/Abstract] OR "Myeloproliferative disorder NOS"[Title/Abstract] OR "Myeloproliferative neoplasms"[Title/Abstract] OR "disease myeloproliferative"[Title/Abstract] OR "diseases myeloproliferative"[Title/Abstract] OR "disorders myeloproliferative"[Title/Abstract] OR "Disease;myeloproliferative"[Title/Abstract] OR "MPD"[Title/Abstract] OR "MPN"[Title/Abstract] OR "Myeloproliferative disease, unspecified"[Title/Abstract] OR "Myeloproliferative disease, NOS"[Title/Abstract] OR ""[Title/Abstract]) OR ("Myeloproliferative disease"[MeSH Terms] OR "Myeloproliferative Disorders"[MeSH Terms] OR "Disorder, Myeloproliferative"[MeSH Terms] OR "Disorders, Myeloproliferative"[MeSH Terms] OR "Myeloproliferative Disorder"[MeSH Terms]) |

**Table20. Literature search strategy of Neuroendocrin cancer**

| Disease area | NEUROENDOCRIN CANCER |
| --- | --- |
| Search date | **2022.12.06.** |
| Search terms | ("Neuroendocrine Tumors"[Title/Abstract] OR "Neuroendocrine Tumor"[Title/Abstract] OR "Tumor, Neuroendocrine"[Title/Abstract] OR "Tumors, Neuroendocrine"[Title/Abstract] OR "Neuroendocrine neoplasm"[Title/Abstract] OR "Neuroendocrine neoplasm (morphologic abnormality)"[Title/Abstract] OR "Neuroendocrine tumor (disorder)"[Title/Abstract] OR "Neuroendocrine tumor (morphologic abnormality)"[Title/Abstract] OR "Neuroendocrine tumour"[Title/Abstract] OR "Neuroendocrine neoplasia"[Title/Abstract] OR "neuroendocrine tumor (diagnosis)"[Title/Abstract] OR "neuroendocrine tumours"[Title/Abstract] OR "neoplasms neuroendocrine"[Title/Abstract] OR "Neuroendocrine tumor NOS"[Title/Abstract] OR ""[Title/Abstract]) OR ("Neuroendocrine Tumors"[MeSH Terms] OR "Neuroendocrine Tumor"[MeSH Terms] OR "Tumor, Neuroendocrine"[MeSH Terms] OR "Tumors, Neuroendocrine"[MeSH Terms]) |

**Table 21. Literature search strategy of Pancreatic cancer**

| Disease area | PANCREATIC CANCER |
| --- | --- |
| Search date | **2022.12.06.** |
| Search terms | ("Malignant neoplasm of pancreas"[Title/Abstract] OR "Cancer of Pancreas"[Title/Abstract] OR "Pancreatic Cancer"[Title/Abstract] OR "Cancer of the Pancreas"[Title/Abstract] OR "Pancreas Cancer"[Title/Abstract] OR "Cancers, Pancreas"[Title/Abstract] OR "Pancreas Cancers"[Title/Abstract] OR "Cancer, Pancreatic"[Title/Abstract] OR "Cancers, Pancreatic"[Title/Abstract] OR "Pancreatic Cancers"[Title/Abstract] OR "Cancer, Pancreas"[Title/Abstract] OR "Malignant tumor of pancreas"[Title/Abstract] OR "Malignant tumor of pancreas (disorder)"[Title/Abstract] OR "CA - Pancreatic cancer"[Title/Abstract] OR "CA - Cancer of pancreas"[Title/Abstract] OR "Malignant tumour of pancreas"[Title/Abstract] OR "Malignant neoplasm of pancreas, part unspecified"[Title/Abstract] OR "Pancreas neoplasm malignant"[Title/Abstract] OR "Malignant neoplasm of pancreas, unspecified"[Title/Abstract] OR "malignant neoplasm of pancreas (diagnosis)"[Title/Abstract] OR "malignant pancreatic neoplasm"[Title/Abstract] OR "Malignant Neoplasm of the Pancreas"[Title/Abstract] OR "pancreas ca"[Title/Abstract] OR "Malignant neoplasm pancreas"[Title/Abstract] OR "malignant neosplasm of the pancreas"[Title/Abstract] OR "Neoplasm malig;pancreas"[Title/Abstract] OR "Pancreas--Cancer"[Title/Abstract] OR "Malignant neoplasm of pancreas, NOS"[Title/Abstract] OR "PANCREAS CANCER MALIGNANT"[Title/Abstract]) OR ("Cancer of Pancreas"[MeSH Terms] OR "Pancreatic Cancer"[MeSH Terms] OR "Cancer of the Pancreas"[MeSH Terms] OR "Pancreas Cancer"[MeSH Terms] OR "Cancers, Pancreas"[MeSH Terms] OR "Pancreas Cancers"[MeSH Terms] OR "Cancer, Pancreatic"[MeSH Terms] OR "Cancers, Pancreatic"[MeSH Terms] OR "Pancreatic Cancers"[MeSH Terms] OR "Cancer, Pancreas"[MeSH Terms]) |

**Table 22. Literature search strategy of Paroxysmal nocturnal haemoglobinuria**

| Disease area | PAROXYSMAL NOCTURNAL HAEMOGLOBINURIA |
| --- | --- |
| Search date | **2022.12.06.** |
| Search terms | ("Paroxysmal nocturnal hemoglobinuria"[Title/Abstract] OR "Marchiafava-Micheli Syndrome"[Title/Abstract] OR "Paroxysmal Hemoglobinuria, Nocturnal"[Title/Abstract] OR "Nocturnal Paroxysmal Hemoglobinuria"[Title/Abstract] OR "Hemoglobinuria, Nocturnal Paroxysmal"[Title/Abstract] OR "Hemoglobinuria, Paroxysmal Nocturnal"[Title/Abstract] OR "Marchiafava Micheli Syndrome"[Title/Abstract] OR "Syndrome, Marchiafava-Micheli"[Title/Abstract] OR "Paroxysmal nocturnal hemoglobinuria (disorder)"[Title/Abstract] OR "PNH"[Title/Abstract] OR "PNH - Paroxysmal nocturnal hemoglobinuria"[Title/Abstract] OR "Paroxysmal nocturnal haemoglobinuria"[Title/Abstract] OR "PNH - Paroxysmal nocturnal haemoglobinuria"[Title/Abstract] OR "Paroxysmal nocturnal hemoglobinuria [Marchiafava-Micheli]"[Title/Abstract] OR "Paroxysmal nocturnal haemoglobinuria [Marchiafava-Micheli]"[Title/Abstract] OR "Paroxysmal nocturnal hemoglobinuria (PNH)"[Title/Abstract] OR "paroxysmal nocturnal hemoglobinuria (diagnosis)"[Title/Abstract] OR "Paroxysmal Hemoglobinuria"[Title/Abstract] OR "Marchiafava-Micheli"[Title/Abstract] OR "hemoglobinuria; paroxysmal, nocturnal"[Title/Abstract] OR "paroxysmal; hemoglobinuria, nocturnal"[Title/Abstract] OR "Marchiafava-Micheli disease"[Title/Abstract] OR "Marchiafava Micheli disease"[Title/Abstract] OR "HEMOGLOBINURIA, PAROXYSMAL, NOCTURNAL"[Title/Abstract] OR "HEMOGLOBINURIA PAROXYSMAL NOCTURNAL"[Title/Abstract]) OR ("Paroxysmal nocturnal hemoglobinuria"[MeSH Terms] OR "Marchiafava-Micheli Syndrome"[MeSH Terms] OR "Paroxysmal Hemoglobinuria, Nocturnal"[MeSH Terms] OR "Nocturnal Paroxysmal Hemoglobinuria"[MeSH Terms] OR "Hemoglobinuria, Nocturnal Paroxysmal"[MeSH Terms] OR "Hemoglobinuria, Paroxysmal Nocturnal"[MeSH Terms] OR "Marchiafava Micheli Syndrome"[MeSH Terms] OR "Syndrome, Marchiafava-Micheli"[MeSH Terms]) |

**Table 23. Literature search strategy of Prostate cancer**

| Disease area | PROSTATE CANCER |
| --- | --- |
| Search date | **2022.12.07.** |
| Search terms | ("Malignant neoplasm of prostate"[Title/Abstract] OR "Prostate Cancer"[Title/Abstract] OR "Prostatic Cancer"[Title/Abstract] OR "Cancer of Prostate"[Title/Abstract] OR "Cancer of the Prostate"[Title/Abstract] OR "Cancer, Prostate"[Title/Abstract] OR "Cancers, Prostate"[Title/Abstract] OR "Prostate Cancers"[Title/Abstract] OR "Cancer, Prostatic"[Title/Abstract] OR "Cancers, Prostatic"[Title/Abstract] OR "Prostatic Cancers"[Title/Abstract] OR "Malignant tumor of prostate"[Title/Abstract] OR "Malignant tumor of prostate (disorder)"[Title/Abstract] OR "Malignant prostatic tumor"[Title/Abstract] OR "CA - Cancer of prostate"[Title/Abstract] OR "Malignant tumour of prostate"[Title/Abstract] OR "Malignant prostatic tumour"[Title/Abstract] OR "Prostatic neoplasms malignant"[Title/Abstract] OR "Prostate cancer NOS"[Title/Abstract] OR "malignant neoplasm of prostate gland"[Title/Abstract] OR "malignant neoplasm of prostate gland (diagnosis)"[Title/Abstract] OR "prostate cancer (diagnosis)"[Title/Abstract] OR "prostate gland neoplasm malignant"[Title/Abstract] OR "Malignant Prostate Neoplasm"[Title/Abstract] OR "Malignant Neoplasm of the Prostate"[Title/Abstract] OR "Malignant Prostate Tumor"[Title/Abstract] OR "Malignant Tumor of the Prostate"[Title/Abstract] OR "Malignant neoplasm prostate"[Title/Abstract] OR "malignant neosplasm of the prostate"[Title/Abstract] OR "Neoplasm malig;prostate"[Title/Abstract] OR "Prostate--Cancer"[Title/Abstract] OR "prostate ca"[Title/Abstract] OR ""[Title/Abstract]) OR ("Prostate Cancer"[MeSH Terms] OR "Prostatic Cancer"[MeSH Terms] OR "Cancer of Prostate"[MeSH Terms] OR "Cancer of the Prostate"[MeSH Terms] OR "Cancer, Prostate"[MeSH Terms] OR "Cancers, Prostate"[MeSH Terms] OR "Prostate Cancers"[MeSH Terms] OR "Cancer, Prostatic"[MeSH Terms] OR "Cancers, Prostatic"[MeSH Terms] OR "Prostatic Cancers"[MeSH Terms]) |

**Table 24. Literature search strategy of Sarcoma**

| Disease area | SARCOMA |
| --- | --- |
| Search date | **2022.12.06.** |
| Search terms | ("Sarcoma"[Title/Abstract] OR "Sarcoma, Soft Tissue"[Title/Abstract] OR "Sarcomas"[Title/Abstract] OR "Sarcomas, Soft Tissue"[Title/Abstract] OR "Soft Tissue Sarcoma"[Title/Abstract] OR "Soft Tissue Sarcomas"[Title/Abstract] OR "Sarcoma (disorder)"[Title/Abstract] OR "Sarcoma (morphologic abnormality)"[Title/Abstract] OR "Malignant mesenchymal tumor"[Title/Abstract] OR "Malignant soft tissue tumor"[Title/Abstract] OR "Malignant mesenchymal tumour"[Title/Abstract] OR "Malignant soft tissue tumour"[Title/Abstract] OR "Cancer of connective tissue"[Title/Abstract] OR "Malignant connective tissue tumor"[Title/Abstract] OR "Malignant connective tissue tumour"[Title/Abstract] OR "Sarcoma NOS"[Title/Abstract] OR "sarcoma (diagnosis)"[Title/Abstract] OR "Sarcoma of Soft Tissue and Bone"[Title/Abstract] OR "Sarcoma of the Soft Tissue and Bone"[Title/Abstract] OR "Soft part sarcoma"[Title/Abstract] OR "Sarcoma, NOS"[Title/Abstract] OR "Soft tissue tumor, malignant"[Title/Abstract] OR "Mesenchymal tumor, malignant"[Title/Abstract] OR "Mesenchymal tumour, malignant"[Title/Abstract] OR "Soft tissue tumour, malignant"[Title/Abstract]) OR ("Sarcoma"[MeSH Terms] OR "Sarcoma, Soft Tissue"[MeSH Terms] OR "Sarcomas"[MeSH Terms] OR "Sarcomas, Soft Tissue"[MeSH Terms] OR "Soft Tissue Sarcoma"[MeSH Terms] OR "Soft Tissue Sarcomas"[MeSH Terms]) |

**Table 25. Literature search strategy of Sickle cell disease**

| Disease area | SICKLE CELL DISEASE |
| --- | --- |
| Search date | **2022.12.06.** |
| Search terms | ("Anemia, Sickle Cell"[Title/Abstract] OR "Sickle Cell Disease"[Title/Abstract] OR "Hemoglobin S Disease"[Title/Abstract] OR "Sickle Cell Disorders"[Title/Abstract] OR "Sickling Disorder Due to Hemoglobin S"[Title/Abstract] OR "HbS Disease"[Title/Abstract] OR "Sickle Cell Anemia"[Title/Abstract] OR "Anemias, Sickle Cell"[Title/Abstract] OR "Sickle Cell Anemias"[Title/Abstract] OR "Disease, Hemoglobin S"[Title/Abstract] OR "Hemoglobin S Diseases"[Title/Abstract] OR "Cell Disease, Sickle"[Title/Abstract] OR "Cell Diseases, Sickle"[Title/Abstract] OR "Cell Disorder, Sickle"[Title/Abstract] OR "Cell Disorders, Sickle"[Title/Abstract] OR "Sickle Cell Diseases"[Title/Abstract] OR "Sickle Cell Disorder"[Title/Abstract] OR "Sickle cell-hemoglobin SS disease"[Title/Abstract] OR "Sickling disorder due to hemoglobin S (disorder)"[Title/Abstract] OR "Sickle cell-hemoglobin SS disease (disorder)"[Title/Abstract] OR "Hemoglobin S-S disease"[Title/Abstract] OR "Hb S disease"[Title/Abstract] OR "Sickle cell syndrome"[Title/Abstract] OR "Drepanocythemia"[Title/Abstract] OR "Hereditary hemoglobinopathy disorder homozygous for hemoglobin S"[Title/Abstract] OR "Hb SS disease"[Title/Abstract] OR "Sickling disorder due to haemoglobin S"[Title/Abstract] OR "Sickle cell-haemoglobin SS disease"[Title/Abstract] OR "Drepanocythaemia"[Title/Abstract] OR "Sickle cell anaemia"[Title/Abstract] OR "Haemoglobin S disease"[Title/Abstract] OR "Haemoglobin S-S disease"[Title/Abstract] OR "Hereditary haemoglobinopathy disorder homozygous for haemoglobin S"[Title/Abstract] OR "Sickle-cell anaemia, unspecified"[Title/Abstract] OR "Sickle-cell anemia"[Title/Abstract] OR "Anaemia sickle cell"[Title/Abstract] OR "Sickle-cell anaemia"[Title/Abstract] OR "Anemia sickle cell"[Title/Abstract] OR "Sickle-cell anemia, unspecified"[Title/Abstract] OR "Sickle cell disease NOS"[Title/Abstract] OR "sickle cell anemia (diagnosis)"[Title/Abstract] OR "sickle cell-hemoglobin SS disease (diagnosis)"[Title/Abstract] OR "anemia hemolytic sickle Hb-SS disease"[Title/Abstract] OR "anemia hemolytic sickle cell anemia"[Title/Abstract] OR "Hb-SS disease"[Title/Abstract] OR "sicklemia"[Title/Abstract] OR "anemia cell disorder sickle"[Title/Abstract] OR "anemia sickle celled"[Title/Abstract] OR "anaemia cell sickle"[Title/Abstract] OR "anemia cells sickles"[Title/Abstract] OR "anemia cells sickle"[Title/Abstract] OR "anemia sickle-cell"[Title/Abstract] OR "sickle-cell disease"[Title/Abstract] OR "anemia cell disorders sickle"[Title/Abstract] OR "cell diseases sickle"[Title/Abstract] OR "cell disorder sickle"[Title/Abstract] OR "cell sickle syndromes"[Title/Abstract] OR "cell sickle syndrome"[Title/Abstract] OR "cells disease sickle"[Title/Abstract] OR "disease hb s"[Title/Abstract] OR "disease sickle cell"[Title/Abstract] OR "disease sickle-cell"[Title/Abstract] OR "scds"[Title/Abstract] OR "syndrome sickle cell"[Title/Abstract] OR "Hemoglobin SS Disease"[Title/Abstract] OR "Herrick"[Title/Abstract] OR "sickle-cell; hemoglobin disease"[Title/Abstract] OR "anemia; Herrick"[Title/Abstract] OR "sickle-cell; anemia"[Title/Abstract] OR "anemia; sickle-cell"[Title/Abstract] OR "Herrick; anemia"[Title/Abstract] OR "sickle-cell; disorder"[Title/Abstract] OR "Anaemia;sickle cell"[Title/Abstract] OR "Sickle-cell disease, unspecified"[Title/Abstract] OR "Sickle-cell disorders"[Title/Abstract] OR "Sickle-cell anemia NOS"[Title/Abstract] OR "Sickle-cell disease NOS"[Title/Abstract] OR "Sickle-cell disorder NOS"[Title/Abstract] OR "SCD"[Title/Abstract] OR "HERRICK SYNDROME"[Title/Abstract] OR "SICKLE CELL HEMOLYTIC ANEMIA"[Title/Abstract] OR "Herrick's anemia"[Title/Abstract] OR ""[Title/Abstract]) OR ("Anemia, Sickle Cell"[MeSH Terms] OR "Sickle Cell Disease"[MeSH Terms] OR "Hemoglobin S Disease"[MeSH Terms] OR "Sickle Cell Disorders"[MeSH Terms] OR "Sickling Disorder Due to Hemoglobin S"[MeSH Terms] OR "HbS Disease"[MeSH Terms] OR "Sickle Cell Anemia"[MeSH Terms] OR "Anemias, Sickle Cell"[MeSH Terms] OR "Sickle Cell Anemias"[MeSH Terms] OR "Disease, Hemoglobin S"[MeSH Terms] OR "Hemoglobin S Diseases"[MeSH Terms] OR "Cell Disease, Sickle"[MeSH Terms] OR "Cell Diseases, Sickle"[MeSH Terms] OR "Cell Disorder, Sickle"[MeSH Terms] OR "Cell Disorders, Sickle"[MeSH Terms] OR "Sickle Cell Diseases"[MeSH Terms] OR "Sickle Cell Disorder"[MeSH Terms]) |

**Table 26. Literature search strategy of Thalassemia**

| Disease area | THALASSEMIA |
| --- | --- |
| Search date | **2022.12.06.** |
| Search terms | (Thalassemias OR "Thalassemia syndrome"[Title/Abstract] OR "Thalassemia (disorder)"[Title/Abstract] OR "Thalassemia syndrome (disorder)"[Title/Abstract] OR "Hereditary leptocytosis"[Title/Abstract] OR "Thalassaemia"[Title/Abstract] OR "Thalassaemia syndrome"[Title/Abstract] OR "Thalassaemic disorders"[Title/Abstract] OR "Thalassaemia NOS"[Title/Abstract] OR "Thalassemia NOS"[Title/Abstract] OR "Thalassemia, unspecified"[Title/Abstract] OR "Thalassaemia, unspecified"[Title/Abstract] OR "hypochromic / microcytic thalassemia"[Title/Abstract] OR "thalassemia (diagnosis)"[Title/Abstract] OR "hypochromic / microcytic thalassemia (diagnosis)"[Title/Abstract] OR "hypochromic / microcytic thalassemia anemia"[Title/Abstract] OR "thalassaemias"[Title/Abstract] OR "syndromes thalassemia"[Title/Abstract] OR "thalassemia disorder"[Title/Abstract] OR "hemoglobinopathy; with thalassemia"[Title/Abstract] OR "anemia; thalassemia"[Title/Abstract] OR "thalassemia; disorder, hemoglobin"[Title/Abstract] OR "thalassemia; hemoglobinopathy (with thalassemia)"[Title/Abstract] OR "leptocytosis; hereditary"[Title/Abstract] OR "thalassemia; anemia"[Title/Abstract] OR "Thalassemia, NOS"[Title/Abstract] OR "Thalassemia syndrome, NOS"[Title/Abstract] OR "Thalassemic syndrome"[Title/Abstract] OR "Thalassemic Syndromes"[Title/Abstract] OR ""[Title/Abstract]) OR ("Thalassemia"[MeSH Terms] OR "Thalassemias"[MeSH Terms]) |

**Table 27. Literature search strategy of Thyroid cancer**

| Disease area | THYROID CANCER |
| --- | --- |
| Search date | **2022.12.06.** |
| Search terms | ("Malignant neoplasm of thyroid"[Title/Abstract] OR "Malignant tumor of thyroid gland"[Title/Abstract] OR "Malignant tumor of thyroid gland (disorder)"[Title/Abstract] OR "Thyroid cancer"[Title/Abstract] OR "Thyroid Ca"[Title/Abstract] OR "Malignant tumour of thyroid gland"[Title/Abstract] OR "Thyroid neoplasms malignant"[Title/Abstract] OR "Malignant neoplasm of thyroid gland"[Title/Abstract] OR "Thyroid gland cancer"[Title/Abstract] OR "Thyroid neoplasm malignant"[Title/Abstract] OR "malignant neoplasm of thyroid gland (diagnosis)"[Title/Abstract] OR "malignant thyroid neoplasm"[Title/Abstract] OR "Malignant Thyroid Gland Neoplasm"[Title/Abstract] OR "Malignant Tumor of the Thyroid"[Title/Abstract] OR "Malignant Neoplasm of the Thyroid"[Title/Abstract] OR "Malignant Neoplasm of the Thyroid Gland"[Title/Abstract] OR "Malignant Thyroid Gland Tumor"[Title/Abstract] OR "Malignant Thyroid Tumor"[Title/Abstract] OR "Malignant Tumor of Thyroid"[Title/Abstract] OR "Malignant Tumor of the Thyroid Gland"[Title/Abstract] OR "cancer of the thyroid"[Title/Abstract] OR "thyroid cancers"[Title/Abstract] OR "Malignant neoplasm thyroid"[Title/Abstract] OR "malignant neosplasm of the thyroid gland"[Title/Abstract] OR "Neoplasm malig;thyroid gland"[Title/Abstract] OR "Cancer of thyroid"[Title/Abstract] OR ""[Title/Abstract]) OR ("Thyroid Neoplasms"[MeSH Terms] OR "Thyroid Cancer, Papillary"[MeSH Terms] OR "Thyroid Nodule"[MeSH Terms]) |

**Table 28. Literature search strategy of Waldenström's Macroglobulinemia**

| Disease area | WALDENSTRÖM'S MACROGLOBULINEMIA |
| --- | --- |
| Search date | **2022.12.06.** |
| Search terms | ("Waldenstrom Macroglobulinemia"[Title/Abstract] OR "Lymphoma, Lymphocytic, Plasmacytoid"[Title/Abstract] OR "Macroglobulinemia"[Title/Abstract] OR "Lymphoma, Lymphoplasmacytoid"[Title/Abstract] OR "Primary Macroglobulinemia"[Title/Abstract] OR "Waldenstrom's Macroglobulinemia"[Title/Abstract] OR "Waldenstrom's Macroglobulinaemia"[Title/Abstract] OR "Macroglobulinemia, Waldenstrom's"[Title/Abstract] OR "Waldenstroms Macroglobulinemia"[Title/Abstract] OR "Macroglobulinemia, Waldenstrom"[Title/Abstract] OR "Macroglobulinaemia, Waldenstrom's"[Title/Abstract] OR "Waldenstrom Macroglobulinaemia"[Title/Abstract] OR "Waldenstroms Macroglobulinaemia"[Title/Abstract] OR "Lymphoplasmacytoid Lymphoma"[Title/Abstract] OR "Lymphomas, Lymphoplasmacytoid"[Title/Abstract] OR "Lymphoplasmacytoid Lymphomas"[Title/Abstract] OR "Macroglobulinemia, Primary"[Title/Abstract] OR "Waldenström macroglobulinemia"[Title/Abstract] OR "Macroglobulinemia (disorder)"[Title/Abstract] OR "Waldenstrom's macroglobulinemia (morphologic abnormality)"[Title/Abstract] OR "Waldenström macroglobulinemia (disorder)"[Title/Abstract] OR "Waldenström macroglobulinaemia"[Title/Abstract] OR "Macroglobulinaemia"[Title/Abstract] OR "Primary macroglobulinaemia"[Title/Abstract] OR "Waldenstrom's macroglobulinaemias"[Title/Abstract] OR "Von Waldenstrom macroglobulinemia"[Title/Abstract] OR "Waldenstrom's macroglobulinaemia NOS"[Title/Abstract] OR "Waldenstrom's macroglobulinemia NOS"[Title/Abstract] OR "Macroglobulinaemia NOS"[Title/Abstract] OR "Von Waldenstrom macroglobulinaemia"[Title/Abstract] OR "Macroglobulinemia NOS"[Title/Abstract] OR "Waldenstrom's macroglobulinemia (diagnosis)"[Title/Abstract] OR "plasmacytoma, macroglobulinemia"[Title/Abstract] OR "lymphoplasmacytic lymphoma"[Title/Abstract] OR "Lymphoplasmacytic Lymphoma/Waldenström Macroglobulinemia"[Title/Abstract] OR "Waldenström's macroglobulinemia"[Title/Abstract] OR "macroglobulinemia, Waldenström's"[Title/Abstract] OR "Lymphoplasmacytic Lymphoma/Waldenström's Macroglobulinemia"[Title/Abstract] OR "macroglobulinemia waldenstrom's"[Title/Abstract] OR "macroglobulinemia waldenstroms"[Title/Abstract] OR "macroglobulinemia waldenstrom"[Title/Abstract] OR "Waldenström; macroglobulinemia"[Title/Abstract] OR "macroglobulinemia; Waldenström"[Title/Abstract]) OR ("Waldenstrom Macroglobulinemia"[MeSH Terms] OR "Lymphoma, Lymphocytic, Plasmacytoid"[MeSH Terms] OR "Macroglobulinemia"[MeSH Terms] OR "Lymphoma, Lymphoplasmacytoid"[MeSH Terms] OR "Primary Macroglobulinemia"[MeSH Terms] OR "Waldenstrom's Macroglobulinemia"[MeSH Terms] OR "Waldenstrom's Macroglobulinaemia"[MeSH Terms] OR "Macroglobulinemia, Waldenstrom's"[MeSH Terms] OR "Waldenstroms Macroglobulinemia"[MeSH Terms] OR "Macroglobulinemia, Waldenstrom"[MeSH Terms] OR "Macroglobulinaemia, Waldenstrom's"[MeSH Terms] OR "Waldenstrom Macroglobulinaemia"[MeSH Terms] OR "Waldenstroms Macroglobulinaemia"[MeSH Terms] OR "Lymphoplasmacytoid Lymphoma"[MeSH Terms] OR "Lymphomas, Lymphoplasmacytoid"[MeSH Terms] OR "Lymphoplasmacytoid Lymphomas"[MeSH Terms] OR "Macroglobulinemia, Primary"[MeSH Terms]) |
